# A cell-based framework for modeling cardiac mechanics

**DOI:** 10.1007/s10237-022-01660-8

**Published:** 2023-01-05

**Authors:** Åshild Telle, James D. Trotter, Xing Cai, Henrik Finsberg, Miroslav Kuchta, Joakim Sundnes, Samuel T. Wall

**Affiliations:** 1 Department of Computational Physiology, Simula Research Laboratory, Oslo, Norway; 2 Department of High Performance Computing, Simula Research Laboratory, Oslo, Norway; 3 Department of Informatics, University of Oslo, Oslo, Norway

**Keywords:** Cardiac mechanics, Cell geometries, Cardiomyocyte contraction, Intracellular and extracellular mechanics, Microscale modeling

## Abstract

Cardiomyocytes are the functional building blocks of the heart—yet most models developed to simulate cardiac mechanics do not represent the individual cells and their surrounding matrix. Instead, they work on a homogenized tissue level, assuming that cellular and subcellular structures and processes scale uniformly. Here we present a mathematical and numerical framework for exploring tissue-level cardiac mechanics on a microscale given an explicit three-dimensional geometrical representation of cells embedded in a matrix. We defined a mathematical model over such a geometry and parametrized our model using publicly available data from tissue stretching and shearing experiments. We then used the model to explore mechanical differences between the extracellular and the intracellular space. Through sensitivity analysis, we found the stiffness in the extracellular matrix to be most important for the intracellular stress values under contraction. Strain and stress values were observed to follow a normal-tangential pattern concentrated along the membrane, with substantial spatial variations both under contraction and stretching. We also examined how it scales to larger size simulations, considering multicellular domains. Our work extends existing continuum models, providing a new geometrical-based framework for exploring complex cell–cell and cell–matrix interactions.

**Supplementary Information:**

The online version contains supplementary material available at 10.1007/s10237-022-01660-8.

## Introduction

Myocardium contraction is known to be affected both by subcellular (Borbély et al. 2005; Azeloglu and Costa 2010) and extracellular (Qin et al. 2007; Deckx et al. 2019) mechanisms. Cellular geometrical configurations have been demonstrated to be important for key mechanical features (Stein et al. 2011; Humphries et al. 2017). Existing models of cardiac tissue mechanics are usually based on extensive homogenization—useful for many purposes, yet unsuitable for capturing smaller-scale effects and interactions. These are widely used on tissue and organ level, see e.g., Guccione et al. (1991); Holzapfel and Ogden (2009), and have been used extensively for interpreting clinical data (Xi et al. 2012; Sack et al. 2016; Finsberg et al. 2018). One of the limitations of this approach, however, is that intracellular and extracellular processes are assumed to take place everywhere instead of being organized into discrete structures.

The cells and their extracellular material each have unique biochemical constituents and structure (Fomovsky et al. 2010; Avazmohammadi et al. 2019) and can be expected to have different mechanical properties. Furthermore, the force that drives cardiac contraction is only generated within the cells and not in the surrounding matrix. There are models that describe how this force is generated from electromechanical subprocesses on a sarcomere level (Land et al. 2017; Rice et al. 2008). When coupled to spatially resolved models, these are usually assumed to scale homogeneously through the tissue without taking into account the cellular geometries. Higher-resolution imaging techniques have been developed, see e.g., Pinali and Kitmitto (2014); Bensley et al. (2016), making it possible to extract exact geometrical representations of how cells are embedded in the extracellular matrix. To make use of this information in cardiac modeling, there is a need for developing spatially resolved modeling frameworks that explicitly capture both the cells and their surroundings.

Some mechanical continuum-based models that do take into account these explicit geometries have been developed, considering single isolated cells. These are modeled using a hyperelastic and isotropic (Tracqui et al. 2008; Okada et al. 2005; Garcia-Canadilla et al. 2017; Lenarda et al. 2018), or anisotropic material model (Tracqui and Ohayon 2009; Ruiz-Baier et al. 2014; Gizzi et al. 2015). The impact of the cell membrane and the extracellular matrix can be explicitly implemented using a combination of Dirichlet and Neumann boundary conditions (Ruiz-Baier et al. 2014; Gizzi et al. 2015; Lenarda et al. 2018). In Lenarda et al. (2018), cell–cell interactions were investigated by considering two cells connected through a surface representing a gap junction. The study presented in Tracqui et al. (2008) considered a single cell embedded in a substrate, investigating the impact of substrate stiffness on intracellular dynamics. None of these, however, include the extracellular matrix as a part of the domain.

Going somewhat broader, there are models for contractile skeletal cells considering cell–matrix interactions. An analytical model was presented in Sharafi and Blemker (2011), backed up with numerical simulations considering up to nine cells connected in a bundle, including an endomysium layer separating the cells from each other. Circular, elliptical and spherical geometries were used in multiple subsequent models, see e.g., Abhilash et al. (2014); Liang et al. (2016); Sopher et al. (2018); Mann et al. (2019), considering single cells and pairs of cells embedded in different kinds of polymer matrices. These works report that considerable amounts of force transmission occur through shear stresses and prominent force chains. Although all of these models have been developed for general contractile cells, the main findings most likely hold for cardiomyocytes as well.

Like mechanics, cardiac electrophysiology has often been modeled in a homogenized manner. Some of these models have been further refined into models that take into account the cells explicitly represented in the domain, surrounded by the extracellular matrix (Hogues et al. 1992; Stinstra et al. 2010; Tveito et al. 2017). Following the terminology in Tveito et al. (2017), such a model can be referred to as an EMI model—a model that separates the domain into an *extracellular* subdomain, a *membrane* and an *intracellular* subdomain.

In this work, we present the extension and characterization of the model presented in Telle et al. (2021), in which the extracellular and the intracellular subdomains are explicitly represented in the geometry. The main purpose behind this model is to capture interactions arising due to differences in structures and properties on a microscale, considering smaller tissue samples built up from individual cells, which potentially could differ in their geometries, material properties, or contraction dynamics. Here, we use experimental data to parametrize our model, considering stretching and shearing experiments. We were able to capture the full orthogonality of cardiac tissue through the geometry rather than imposing it in the strain energy function. Using this parametrized model as a baseline, we explored the parameter space subject to fiber direction stretch and contraction. Utilizing high-performance computing (HPC), we are able to move from the single cells to multicellular domains, representing small cubical tissue samples.

## Models and methods

### The mathematical framework

**Fig. 1:**
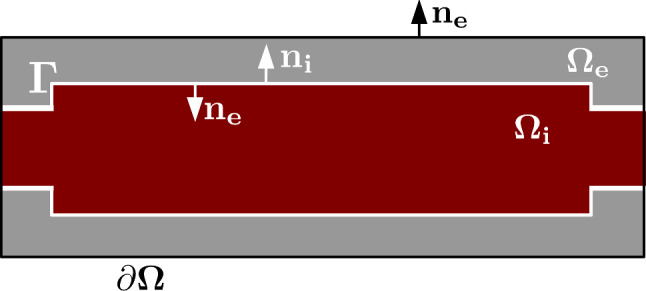
Subdomains $\Omega _i$ and $\Omega _e$ and their boundaries. Schematic drawing of the subdomains; the intracellular subdomain $\Omega _i$ is surrounded by a surface $\Gamma$ representing the membrane, separating it from the extracellular subdomain $\Omega _e$. The whole domain is surrounded by an outer boundary $\partial \Omega$. $\mathbf {n_i}$ and $\mathbf {n_e}$ denote normal vectors of surfaces $\Gamma$ and $\partial \Omega$. In 3D, $\Omega _i$ and $\Omega _e$ are volumes, separated by a surface $\Gamma$

Following the geometrical framework presented in Tveito et al. (2017) and employed in Telle et al. (2021), we consider a three-dimensional domain consisting of two volumes—the cells and the surrounding matrix. The intracellular space ($\Omega _i$) is surrounded by the extracellular space ($\Omega _e$), separated by a surface ($\Gamma$) representing the cell membrane; see Fig. 1. Geometrically $\Omega _i$ and $\Omega _e$ are both closed volumes, while $\Gamma$ represents the intersection $\partial \Omega _i \cap \partial \Omega _e$, where again $\partial \Omega _i$ and $\partial \Omega _e$ represent the boundaries of $\Omega _i$ and $\Omega _e$. We let $\Omega = \Omega _i \cup \Omega _e$ denote the whole domain and $\partial \Omega$ the outer boundary.

Using a fully Lagrangian formulation, let ${\textbf{u}}$ denote the displacement of a given point in the domain $\Omega$, defined by ${\textbf{u}}:= {\textbf{x}} - {\textbf{X}}$, where ${\textbf{x}}$ is a point in the current configuration and ${\textbf{X}}$ a point in the reference configuration. Let ${\textbf{F}}:= \nabla {\textbf{u}} + {\textbf{I}}$ denote the deformation gradient, where ${\textbf{I}}$ is the identity tensor. The stress–strain relationship in the material is given by a strain energy function $\psi ({\textbf{F}})$, and the first Piola-Kirchhoff stress tensor by ${\textbf{P}}:= \frac{\partial \psi ({\textbf{F}})}{\partial {\textbf{F}}}.$ An equilibrium solution, where all forces are balanced, can be found by solving

1 $$\begin{aligned} \nabla \cdot {\textbf{P}} =  0 \end{aligned}$$

over the whole domain $\Omega$, subject to imposed boundary conditions.

We assumed continuity of displacement and stresses across the membrane, mathematically expressed as

2 $$\begin{aligned} \mathbf {u_i} =  \mathbf {u_e} \end{aligned}$$

and

3 $$\begin{aligned} \mathbf {n_i} {\textbf{P}} =  - \mathbf {n_e} {\textbf{P}} \end{aligned}$$

on $\Gamma$ where $\mathbf {n_i}$ and $\mathbf {n_e}$ denote normal vectors of $\Omega _i$ and $\Omega _e$ respectively.

To incorporate contraction of the cells, we used an active strain approach, as described, e.g., in Ambrosi and Pezzuto (2012); Rossi et al. (2012). In this approach, the deformation gradient ${\textbf{F}}$ is decomposed in a multiplicative manner, ${\textbf{F}} = \mathbf {F_p} \mathbf {F_a}$. The active component, $\mathbf {F_a}$, quantifies the strain caused by the cell contraction over time, while $\mathbf {F_p} = {\textbf{F}} \mathbf {F_a}^{-1}$ gives the passive elastic deformation. Following Ruiz-Baier et al. (2014) and Rossi et al. (2012), we let $\mathbf {F_a}$ be transversely isotropic, given by

4 $$\begin{aligned} \mathbf {F_a} = \begin{bmatrix} 1 - \gamma &{} 0 &{} 0 \\ 0 &{} (1 - \gamma )^{-1/2} &{} 0 \\ 0 &{} 0 &{} (1 - \gamma )^{-1/2} \end{bmatrix}, \end{aligned}$$

where $\gamma$ is a scalar time-dependent function.

In our model, active contraction was only prescribed within $\Omega _i$, i.e., within the cells. We imposed the discretization by letting $\gamma$ vary over time within $\Omega _i$ while being set to zero in $\Omega _e$:

5 $$\begin{aligned} \gamma = {\left\{ \begin{array}{ll} \gamma _i (t) \quad \quad \,\,\, {\textbf{X}} \in \Omega _i \\ 0 \qquad \qquad {\textbf{X}} \in \Omega _e. \end{array}\right. } \end{aligned}$$

For simplicity, we took $\gamma _i$ to be a scalar function dependent on time only.

For the strain energy function $\psi$, we defined different strain energy functions $\psi _i$ and $\psi _e$ for the intracellular and extracellular spaces. We then combined these into one common strain energy function,

6 $$\begin{aligned} {\psi ({\textbf{F}})} = {\left\{ \begin{array}{ll} \psi _i({\textbf{F}}) \quad \quad {\textbf{X}} \in \Omega _i \\ \psi _e({\textbf{F}}) \quad \quad {\textbf{X}} \in \Omega _e \end{array}\right. } \end{aligned}$$

uniquely defined in $\Omega \setminus \Gamma$.

For both domains, we used a hyperelastic invariant-based strain energy function, based on the one proposed in Holzapfel and Ogden (2009). These were based on the invariants $I_1$ and $I_{4f}$, given by

7 $$\begin{aligned} I_1: =  \textrm{tr} (\overline{{\textbf{C}}}) \end{aligned}$$

8 $$\begin{aligned} I_{4f}: =  \mathbf {f_0} \cdot (\overline{{\textbf{C}}} \mathbf {f_0}). \end{aligned}$$

Here $\textrm{tr}$ denotes the trace operator, $\overline{{\textbf{C}}}:= J^{-2/3} \mathbf {F_p}^\textrm{T} \mathbf {F_p}$ the modified isochoric Cauchy–Green deformation tensor, and $\mathbf {f_0}$ the fiber direction, i.e., the longitudinal direction of the cells.

For the intracellular subdomain, we let

9 $$\begin{aligned} \psi _{i}({\textbf{F}}) =  \frac{a_i}{2 b_i} (e^{b_i (I_1 - 3)} - 1) \nonumber {}  {} + \frac{a_{if}}{2 b_{if}} (e^{b_{if} \Vert I_{4f} - 1 \Vert _{+}^2} - 1). \end{aligned}$$

Here $\Vert \cdot \Vert _{+}$ denotes a conditional term, given by $\Vert I \Vert _{+} = \textrm{max}(I, 0)$. The parameters $a_i$, $b_i$, $a_{if}$ and $b_{if}$ determine the cellular stiffness. The second component gives significant increasing stiffness in the fiber direction subject to stretching, and none under compression.

For the extracellular domain, we used an isotropic formulation, given by

10 $$\begin{aligned} \psi _{e}({\textbf{F}}) =  \frac{a_e}{2 b_e} (e^{b_e (I_1 - 3)} - 1). \end{aligned}$$

Again, the parameters $a_e$ and $b_e$ determine the stiffness of the matrix surrounding the cells. Other variants for this strain energy functions were also explored. In particular, we tried including shear terms and terms increasing the stiffness in transverse (sheet and normal) directions. These additional terms were, however, found to be redundant.

Cardiac tissue is known to be fully orthotropic, with the sheet direction determined by distinct alternating layers of cells and perimysium (Holzapfel and Ogden 2009; Costa et al. 1999). Rather than imposing this in the strain energy function, we let the full orthotropy of myocardium be imposed by including layers in the geometry used in the simulations—see detailed description in Sect. 2.3.

In this work, we assumed both subdomains to be incompressible, i.e., we required

11 $$\begin{aligned} J:= \textrm{det}({\textbf{F}}) = 1 \end{aligned}$$

for all ${\textbf{X}}$ in $\Omega$ during deformation. This restriction was imposed using a Lagrange multiplier, see full derivation in Holzapfel (2000). This gives us a modified strain energy function,

12 $$\begin{aligned} \psi ^*({\textbf{F}}) =  \psi + p (J -1), \end{aligned}$$

where *p* can be interpreted as the hydrostatic pressure.

### Weak form and implementation details

To solve the above systems of equations numerically, we used the finite element method. Here, we solved (1) in the weak sense by solving the following problem: Find displacement ${\textbf{u}}$ and the hydrostatic pressure *p* such that

13 $$\begin{aligned} \int _{\Omega } {\textbf{P}}: \nabla {\textbf{v}} + q(J - 1) \textrm{d}{\textbf{X}} =  0 \end{aligned}$$

for all test functions ${\textbf{v}}$ and *q* from suitable test spaces. A full derivation can be found in Appendix A.1. The numerical experiments were implemented using FEniCS (Alnæs et al. 2015) (version 2019.1), using a Taylor-Hood discretization ($P_2$–$P_1$) to represent the displacement and the pressure, respectively.

Equation (13) is solved as a stationary problem for each time step. Before we solve the problem for a given step, either boundary displacement (for stretch/shear experiments) or active tension (5) (for contraction experiments) values are updated. The results from the previous step are used as an initial guess for the next step, with zero values being used for the very first step. Each such step can hence be considered a continuation step, for which Newton’s method is used to find an equilibrium solution.

### Geometries and meshes

**Fig. 2:**
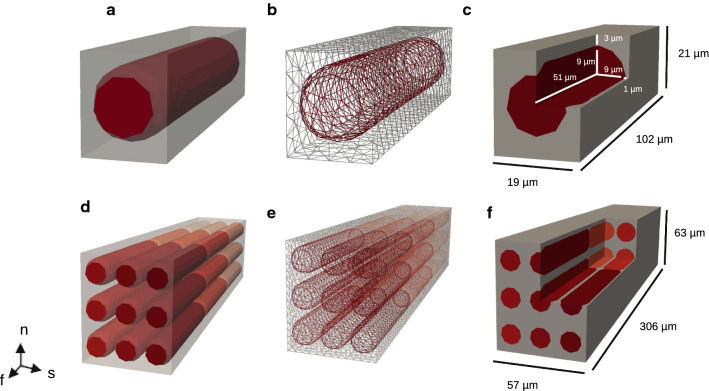
Geometries and meshes. The geometries (**a**, **d**), the corresponding meshes (**b**, **e**), and the geometries with a quarter of the domain removed (**c**, **f**)—representing the single cell and the $3 \times 3 \times 3$ cells. The cell has a cylindrical shape, determining the fiber direction ${\textbf{f}}$, and the membrane is represented as an explicit surface in the mesh. The single-cell mesh was used as a base for the tiled mesh, combined into a common geometry by being tiled in a grid fashion. Note that the padding in the normal direction ${\textbf{n}}$ is larger than the padding in the sheet direction ${\textbf{s}}$, meant to resemble layers of perimysium. The dimensions are indicated in (**c**) and (**f**)

**Table 1:** Properties of meshes used for convergence experiments

Max c	h$_{\textrm{max}}$	h$_{\textrm{min}}$	Nodes	Elements	Dofs
20.0	18.72	4.12	265	1 118	5 560
10.0	17.98	4.37	335	1 364	6 956
5.0	9.66	3.62	965	4 071	20 573
2.5	5.32	2.02	4 117	18 964	91 540
1.25	2.69	1.20	24 362	126 908	574 139

**Table 2:** Properties of meshes used for scaling (HPC) experiments

Cardiac cells	Nodes	Elements	Dofs
1	965	4 071	20 573
2	8 891	8 142	40 702
$2\times 2$	3 515	16 284	78 218
$2 \times 2 \times 2$	6 461	32 568	149 360
$4 \times 2 \times 2$	12 787	65 136	297 082
$4 \times 2 \times 4$	24 601	130 272	582 076
$4 \times 4 \times 4$	47 033	260 544	1 136 588
$8 \times 4 \times 4$	93 565	521 088	2 266 888

For our experiments, we considered meshes representing a single cardiac cell embedded in an extracellular matrix, as well as tiled meshes consisting of multiple cells; see Fig. 2.

We take the fiber direction ${\textbf{f}}_0$ to be aligned with the cells in the longitudinal direction. Following the convention for several tissue-level models, we define sheet and normal directions ${\textbf{s}}_0$ and ${\textbf{n}}_0$, perpendicular to the fiber direction and each other. We follow the usual convention of defining the microstructural unit vectors in the reference configuration using a subscript 0, and the corresponding vector in the current configuration without the subscript.

The cell itself has a cylindrical shape, having a length of 102 $\upmu {\textrm{m}}$ (100 $\upmu {\textrm{m}}$ + 1 $\upmu {\textrm{m}}$ for each of the connections) and a diameter of 18 $\upmu$m, with rounded edges at each end; see Fig. 2 (c, f). The extracellular material was added around to form a box, such that the matrix surrounds the cell, with the thinnest layer being 1 $\upmu {\textrm{m}}$ in the sheet direction and 3 $\upmu {\textrm{m}}$ in the normal direction. The thicker layers in the normal direction were added to resemble layers of perimysium. Together with the cell orientations, these give rise to the characteristic local three-dimensional structure of myocardium. These proportions give us a total volume of $24.42 \cdot 10^3$ $\upmu {\textrm{m}}^3$ for $\Omega _i$, and $24.54 \cdot 10^3$ $\upmu {\textrm{m}}^3$ for $\Omega _e$ for a single-cell geometry.

The regular cubical shape of the domain was explicitly developed for the purpose of extending the framework to multicellular domains representing varying numbers of cardiac cells. Here, copies of the single-cell geometry were simply tiled next to each other in width, length, and height. The cells were connected in the fiber direction, sharing a common surface in the mesh at the connections.

Properties of meshes of various resolutions for a single-cell geometry are reported in Table 1. These were all used for convergence studies, and for most other experiments, the middle (5.0 $\upmu {\textrm{m}}$) mesh was used. Tiled meshes, generated using the 5.0 $\upmu {\textrm{m}}$ as a base, were used for scaling experiments. Properties of these meshes are listed in Table 2. The single-cell mesh (5.0 $\upmu {\textrm{m}}$) can be seen in Fig. 2a–c, and a corresponding tiled mesh is displayed in Fig. 2d–f. All meshes are tetrahedral and were generated using Gmsh (Geuzaine and Remacle 2009).

### Deformation modes

**Fig. 3:**
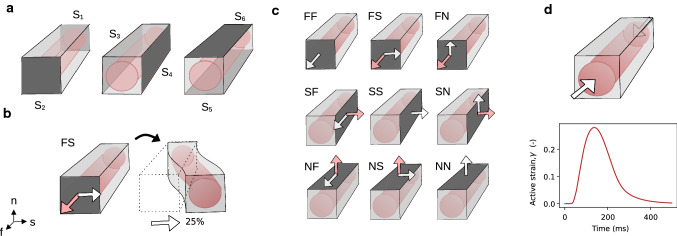
Surface partition, boundary conditions and deformation modes. The outer boundary surface was partitioned into different surfaces (**a**), where ${\partial \Omega }= {S_1} \cup\cdots \cup S_6$. Stretching and shearing experiments were performed by applying Dirichlet boundary conditions for the displacement on either of these pairs of opposite surfaces. In, e.g., shear FS (**b**), points on the surface S_2_ are moved a given distance (in percentage of the length of the domain) in the sheet direction. There are nine deformation modes used for the model parameterization (**c**)—stretching experiments FF, SS, and NN; shearing experiments FS, FN, SF, SN, NF, and NS. The white arrows indicate the direction of deformation. The pink arrows indicate the normal component, when not coinciding. For the active contraction experiments (**d**), all surfaces were allowed to deform freely (top). Active strain was imposed in the intracellular domain Ω_i_, using a precomputed active strain transient (bottom)

Virtual stretching and shearing experiments were used first to parametrize our EMI model. This parameterized model was then used to explore convergence, sensitivity, spatial variation, and scalability. For these simulations, we considered nine distinct deformation modes—stretching in the fiber, sheet and normal directions (FF, SS and NN), and shearing in all combinations of fiber/sheet/normal directions (FS, FN, SF, SN, NF, NS). See Fig. 3c for a schematic overview. For each deformation mode, displacement on pairwise opposite surfaces (see Fig. 3a) was enforced using Dirichlet boundary conditions while the other surfaces were allowed to move freely. A complete mathematical description of all boundary conditions can be found in Appendices A.2 and A.3.

Cellular contraction was modeled using a precomputed time-dependent active strain transient, as displayed in Fig. 3d. For these experiments, the traction along every surface was set to zero. Rigid motion was avoided by enforcing orthogonality of the solution with respect to the kernel using Lagrangian multipliers, as explained, e.g., in Kuchta et al. (2016). See Appendix A.4 for more details.

### Reported quantities

For many experiments, we report values derived from the Green-Lagrange strain tensor, given by

14 $$\begin{aligned} {\textbf{E}} = \frac{1}{2} ({\textbf{F}}^\mathrm{T} {\textbf{F}} - {\textbf{I}}) \end{aligned}$$

and values derived from the Cauchy stress tensor, given by

15 $$\begin{aligned} \mathbf {\sigma } = J^{-1} {\textbf{P}} {\textbf{F}}^\mathrm{T}. \end{aligned}$$

In particular, we considered the normal components along the fiber, sheet, and normal directions of each tensor, i.e., $E_{ff}$, $E_{ss}$ and $E_{nn}$ for the strain, and $\sigma _{ff}$, $\sigma _{ss}$ and $\sigma _{nn}$ for stress. Except from when displayed as spatial plots, these were taken as averaged quantities over the separate subdomains. These were calculated as volume integrals, given by

16 $$\begin{aligned} \overline{E_{ee}} = \frac{\int _{\Omega _j} \mathbf {e_0} \cdot ({\textbf{E}} \mathbf {e_0}) \textrm{d}{\textbf{X}}}{\int _{\Omega j} \textrm{d}{\textbf{X}}} \end{aligned}$$

and

17 $$\begin{aligned} \overline{\sigma _{ee}} = \frac{\int _{\Omega _j} {\textbf{e}}_0 \cdot (\mathbf {\sigma } {\textbf{e}}_0) \textrm{d}{\textbf{X}}}{\int _{\Omega j} \textrm{d}{\textbf{X}}}. \end{aligned}$$

Here $\mathbf {e_0}$ is equal to $\mathbf {f_0}$, $\mathbf {s_0}$ or $\mathbf {n_0}$, in the reference configuration. Subdomain $\Omega _j$ is either the (combined) intracellular space $\Omega _i$, the intracellular space in a single cell ($\Omega _{i, k}$ for $k = 1... N$, considering *N* cells) or the extracellular space $\Omega _e$.

The integrated quantities were computed using fourth-order Gaussian quadrature, which coincided with second and third order for the strain values. For visualization of the spatial distributions, these were projected to a discontinuous Lagrangian function space ($DG_2$), the discontinuity being helpful for capturing strain and stress components close to the membrane.

### Parameter estimation

To parametrize our model, we used the same experimental data as explored in Kakaletsis et al. (2021), made publicly available by the authors (Kakaletsis et al. 2020). In their study, cardiac tissue blocks from sheep hearts were extracted, then shearing and stretching experiments were performed on each sample. We restricted the scope to the 11 samples taken from the left ventricle. In order to make the optimization tractable with our given computation resources, we only considered the positive values, i.e., from reported displacement zero and upwards.

For each sample, there were data on forces, in both normal and shear directions, and resulting displacement, as well as length, width and height measurements for each of the samples. We derived load and stretch values for each sample and used these for direct comparison to the model.

We performed corresponding virtual stretch and shear experiments by deforming the domain using the same range of stretch and shear magnitudes used in the experimental data. For each stretch value $\lambda$ and each deformation mode *M*, the total load on the surface, imposed by the applied Dirichlet boundary conditions, was calculated by

18 $$\begin{aligned} L_M(\lambda , {\textbf{e}}) = \frac{\int _{S_i} {\textbf{P}} {\textbf{N}} \cdot {\textbf{e}} \textrm{dS}}{\int _{S_i} {\textbf{F}} {\textbf{N}} \cdot ({\textbf{F}}^\mathrm{T} {\textbf{N}}) \textrm{dS}}. \end{aligned}$$

Here ${\textbf{e}}$ is either the mesh normal direction or the shear direction, depending on the tracked component. $S_i$ is either $S_2$, $S_4$ or $S_6$, depending on the deformation mode (see again Fig. 3), and ${\textbf{N}}$ denotes the normal vector to this surface.

The parameter estimation was then formulated as an optimization problem, expressing the difference between the experimental load values and the virtual load values as an $L_2$ norm. We performed the optimization using SciPy’s *minimize* function, allowing material parameters $a_i$, $b_i$, $a_e$, $b_e$, $a_{if}$ and $b_{if}$ to vary within the interval [0.01, 40].

#### Parameter sensitivity

We next explored the sensitivity of each parameter by computing Sobol indices. This analysis was done for all deformation modes used in the stretching and shear experiments, tracking load values of interest, as well as for averaged stresses across the entire domain under fiber direction stretch experiment FF and contraction. The sensitivity analysis was performed using the Python library *SALib* (Herman and Usher 2017; Iwanaga et al. 2022).

For each of these cases, sensitivity analysis was performed by sampling the parameter space with $N = 512$ values, allowing each material parameter to vary in the interval [0.1, 30]. We considered $D = 6$ material parameters, which resulted in $N (D + 2) = 512 (6 + 2) = 4096$ different parameter combinations per deformation mode. For each parameter combination, we simulated virtual stretch up to 10% and virtual shear up to 40%, dictated by the range of strain values of the experimental dataset used for the parameterization. Contraction was simulated to a peak of approximately 20% shortening. These experiments were all performed using a single-cell geometry. Finally, based on the resulting load and stress values, we computed the first order and total sensitivity indices.

#### Convergence

To test the convergence properties of our problem, we generated a set of meshes with decreasing maximum mesh element size (circumradius), as listed in Table 1 (Max c). Here h$_{\textrm{max}}$ and h$_{\textrm{min}}$ are defined as the largest and smallest cell diameter in the mesh. The cell diameter is defined as twice the circumradius.

We performed convergence experiments by tracking normal stress components for each subdomain, as given in (17), considering fiber direction stretch and contraction. These were also performed using a single-cell geometry.

### Spatial plots

To explore spatial distributions we performed fiber direction stretch and contraction experiments, processed in Paraview. The fiber direction stretch was simulated up to 10% stretch. For the contraction experiment, we simulated half a cardiac cycle—and report spatial values for the maximal contracted state (138 ms). For the contraction experiments, we also compared individual components of stress and strain values averaged over each subdomain. We considered both single cell meshes and tiled meshes, consisting of $3 \times 3 \times 3$ cells.

### Scalability

Finally, we investigated the performance and scalability of our current solver implementation with the aim of tackling larger, tissue-scale problems using meshes of tiled cells. For these experiments, we stretched the domain by 15 % in the fiber direction using ten continuation steps while varying the problem size and number of CPUs used to perform the calculations. We considered one weak scaling problem, where we used tiled meshes such that there is one cardiac cell per CPU, and one strong scaling problem, where a fixed mesh representing $4\times {}4\times {}4$ cells was used. Properties of meshes used in these experiments are listed in Table 2.

The current solver is based on using Newton’s method combined with a distributed-memory parallel direct solver provided by SuperLU_dist (Li and Demmel 2003). A known limitation of this approach is the considerable memory usage that is associated with the LU factorization stage of the direct solver (see, e.g., Dongarra et al. (1998)). To increase the amount of available memory, in order to make the problem feasible to solve, we employed six compute nodes for the strong scaling experiments. Each compute node consisted of two Intel Xeon Gold 6138 CPUs with 40 CPU cores and 192 GiB of memory. Consequently, we limited our current experiments to at most 240 CPU cores and a total of $1\,152$ GB of memory. We configured SuperLU_dist to use the serial MC64 algorithm to compute a row permutation. An alternative parallel algorithm for based on Approximate Weight Perfect Matching (AWPM) (Azad et al. 2020) was tested, but found to be significantly slower for our case.

## Results

### Parameter estimation

**Table 3:** Optimized material parameters for the strain energy function (6) to experimental data (Kakaletsis et al. 2020), for samples 1–11

	$a_i$	$b_i$	$a_e$	$b_e$	$a_{if}$	$b_{if}$
Sample 1	0.02	22.44	8.15	6.84	40.00	24.16
Sample 2	29.51	14.49	0.57	2.29	40.00	40.00
Sample 3	4.31	17.06	0.18	16.39	7.66	15.11
Sample 4	0.02	5.99	3.76	5.90	21.21	16.91
Sample 5	3.15	1.92	0.03	37.47	40.00	16.62
Sample 6	12.25	0.01	0.05	31.30	17.34	8.16
Sample 7	3.06	23.57	0.04	17.03	9.46	27.16
Sample 8	2.86	0.01	0.01	14.93	5.95	27.63
Sample 9	3.20	24.46	0.02	25.45	30.62	17.21
Sample 10	3.17	18.18	0.05	11.97	3.76	38.98
Sample 11	1.16	0.27	3.87	9.87	2.16	40.00
**Average**	**5**.**70**	**11**.**67**	**1**.**52**	**16**.**31**	**19**.**83**	**24**.**72**

The average values are highlighted in bold

**Fig. 4:**
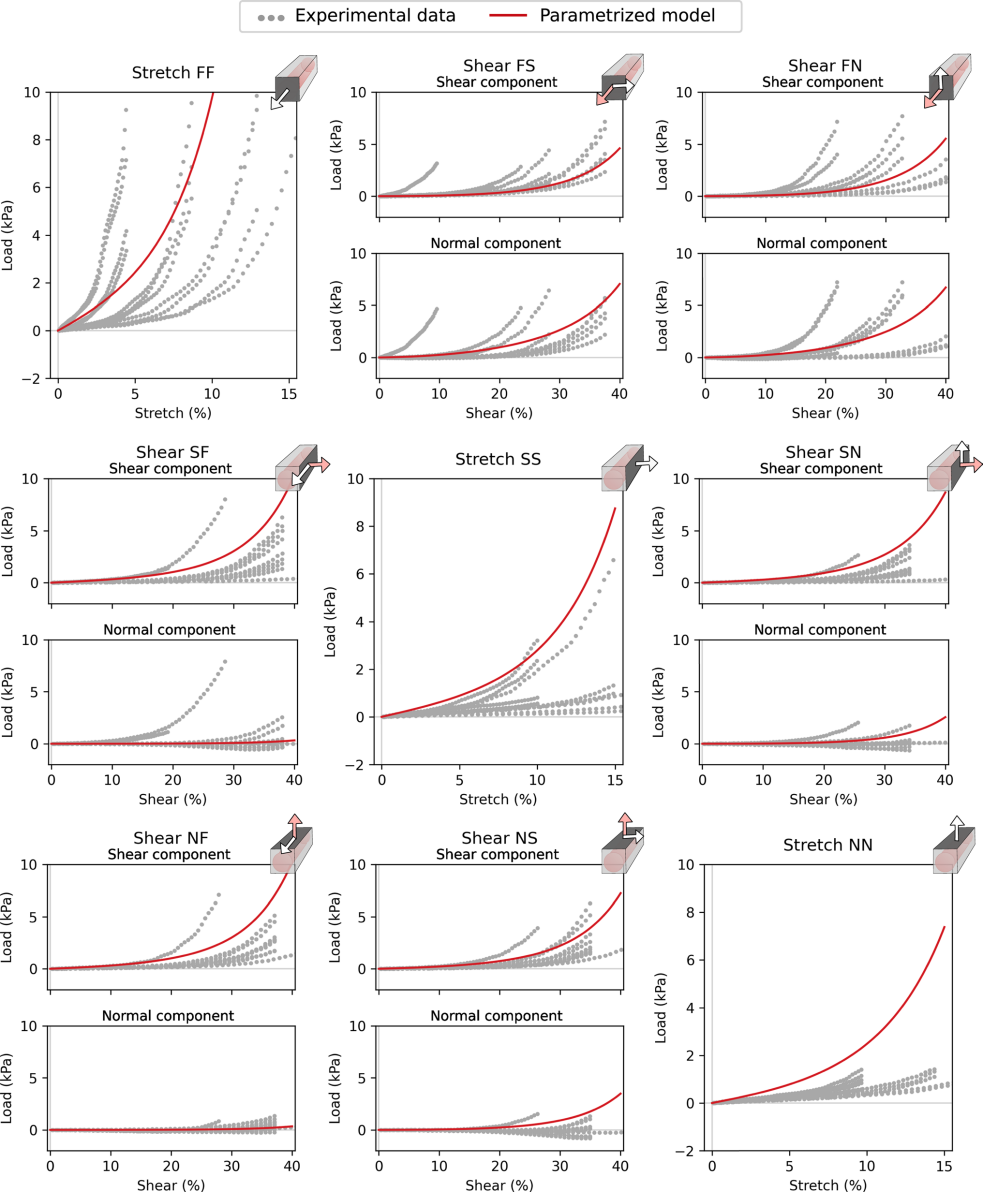
Parameter fit—all experimental data and our parametrized model. Original experimental data for all deformation modes FF–NN shown with gray dots. Our model fit for the same deformation modes, using average material parameter values as listed in Table 3, is displayed in red. The schematic drawings (top right corners) display displacement direction (white), normal (pink/white) and shear (white) components; see more detailed explanation in Fig. 3

Optimized parameter values for (9) and (10) found for each experimental sample, as well as the average across all of them, are listed in Table 3. The experimental data are plotted together with the model fit, using average parameters, in Fig. 4. We observe that most of the load values fall within the range given by the experimental data—in particular, the load values calculated for the FF, FS, and FN experiments all are close to the middle of the range defined by the experimental data. On the other hand, the load values for the sheet and normal direction stretch (SS, NN) are both somewhat too high.

#### Parameter sensitivity

**Fig. 5:**
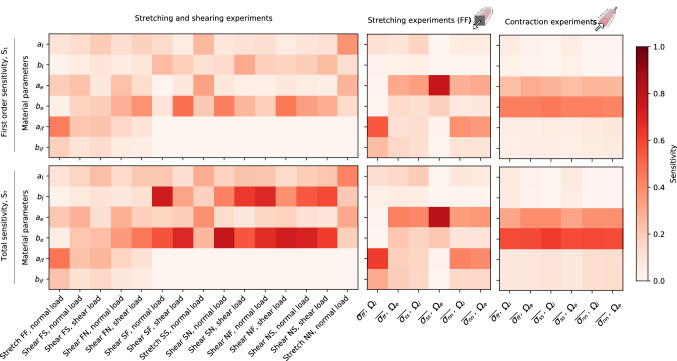
Sensitivity analysis. Sensitivity analysis performed using Sobol indices, reporting first order ($S_1$, top) and total ($S_T$, bottom) sensitivities. The sensitivity analysis was performed for all stretching and shear experiments with normal and shear loads as output (left), as well as fiber direction stretch and contraction with normal intra- and extra-cellular stresses as output (middle, right)

**Fig. 6:**
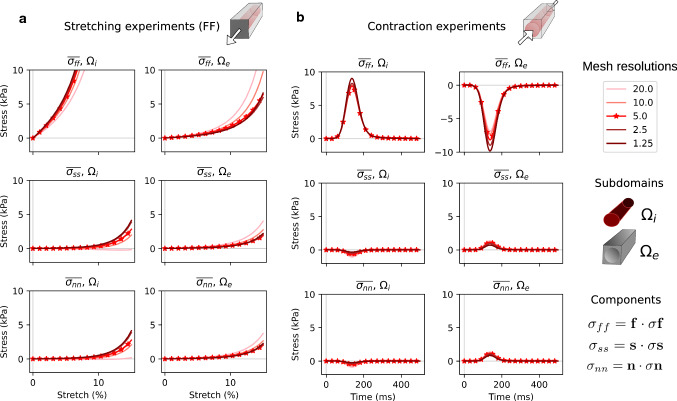
Convergence experiments—normal stress components. The plots display the effect of mesh refinement (see Table 1), measured by the change in integrated stress values subject to fiber direction stretch (**a**) and contraction (**b**). The lightest line corresponds to the coarsest mesh (20.0 μm), while the darkest line corresponds to the finest mesh (1.25 μm). For both deformation modes we display the normal stress components of $\mathbf {\sigma }$ (15) averaged over intracellular subdomain Ω_i_ and the extracellular subdomain Ω_e_, respectively, as given by (17). The curves marked with stars display the resolution used for most of the experiments in this paper

Sensitivity analysis was performed using variance-based sensitivity analysis, with the resulting Sobol indices displayed in Fig. 5. We here report the first order ($S_1$) and total ($S_T$) sensitivity, and for the sake of brevity we do not show the confidence intervals. Overall, the confidence intervals span a range up to 0.23 for the load experiments, up to 0.09 for the fiber direction stress experiments, and up to 0.12 for the contraction stress experiments. Across all experiments, we see that the general patterns of the first-order sensitivity are repeated in the total sensitivity, and more so for the averaged stress values than for the load values.

For the load experiments, we see that most of the modes are most sensitive to changes in the exponential $b_e$ parameter, followed by the exponential $b_i$ parameter. Prominently, the exceptions are the three stretch experiments, which, in particular, have low sensitivity for these two parameters. Instead, the $a_{if}$ parameter appears to matter most for the fiber direction stretch (FF), the $a_i$ and $a_e$ parameters most for the sheet direction stretch (SS), and $a_i$ followed by $a_e$ for the normal direction stretch (NN).

Considering the fiber direction stress experiment, we can see that the intracellular fiber direction stress ($\overline{{\sigma }_{ff}}$ across $\Omega _i$) is most sensitive to the parameter $a_{if}$, as for the corresponding load experiment. The extracellular sheet direction stress ($\overline{{\sigma }_{ss}}$ across $\Omega _e$) is by far most sensitive to the $a_i$ parameter. Across all tracked stresses, the $b_i$ parameter matters the least.

Finally, for the contraction stress experiment, across all tracked stresses, the $b_e$ parameter matters the most, seconded by $a_e$. Across the other parameters, the sensitivity is, in comparison, marginal. This holds for both the first order and the total sensitivity, although we can see a small enhancement in all values for the total sensitivity, i.e., having somewhat higher magnitudes.

#### Convergence

Results from the convergence studies are displayed in Fig. 6, reporting subdomain stress values for different mesh resolutions. Many of the tracked components appear converged at a mesh resolution with a maximum element size of 5.0 $\upmu {\textrm{m}}$. However, under contraction, component $\sigma _{ff}$ appears to still vary somewhat going from the mesh with maximum element size of 2.5 $\upmu {\textrm{m}}$ to the mesh with an element size of 1.25 $\upmu {\textrm{m}}$.

**Fig. 7:**
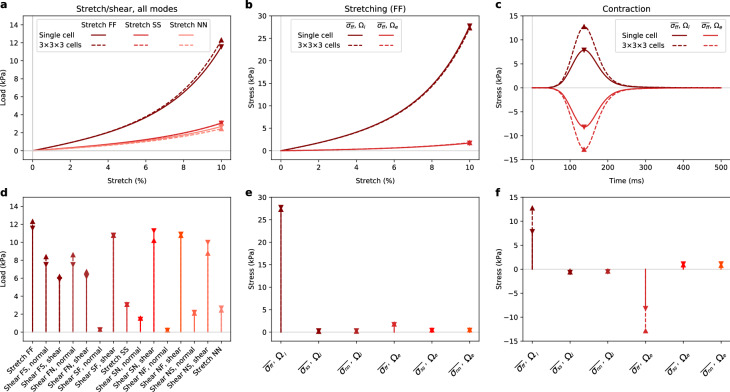
Single cell vs tiled cells behavior, loads and stresses. Representative plots on the top, all modes at peak values (indicated by triangles) on the bottom. We also compared intra- and extracellular stresses along the fiber direction, under fiber direction stretch (FF; **b**, **d**) and under contraction (**c**, **e**)

**Fig. 8:**
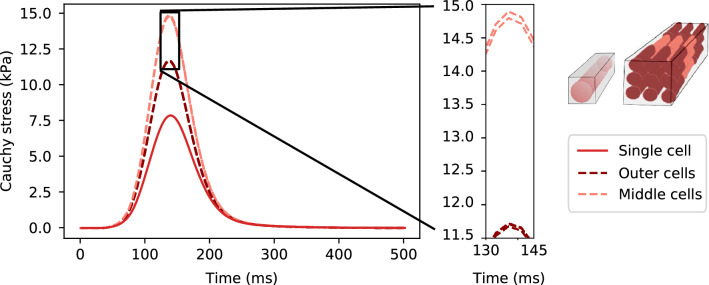
Cardiomyocyte stress per cell, single cell and $3 \times 3 \times 3$ cells. Average Cauchy stress in the fiber direction, $\overline{\sigma _{ff}}$ (17), for a single cell (solid curve) and for each cell in a multicellular domain (dashed curves). For the tiled cells, we use a lighter color for the middle cells, relative to the fiber direction, as indicated by the schematic drawing. The magnified area displays minor differences within each of these groups

#### Single cell versus multicellular domains

We compared the load and stress results for a single cell, which was used for the parametrization, and $3 \times 3 \times 3$ cells. The results are displayed in Fig. 7. We note that across all stretching and shearing experiments, considering load values, the results are similar for both geometries. For stresses in the stretching experiment (subplots b, e), most quantities are comparable (in absolute values; relative values vary somewhat more), while for contraction, there is a notable difference. Here, the averaged fiber direction stress given by $\overline{{\sigma }_{ff}}$ across $\Omega _i$, almost doubles from 7.87 kPa at peak for the single-cell domain to 12.75 kPa for the multicellular domain. Similarly, but reversed in magnitude, the fiber direction stresses given by $\overline{{\sigma }_{ff}}$ across $\Omega _e$, decreases from −8.17 kPa at peak for the single-cell domain to −12.85 kPa for the multicellular domain. The individual contributions of the fiber direction stresses of each cell are also plotted in Fig. 8. Here, we can see that the fiber direction stresses are higher in all cells in the tiled mesh compared to the single-cell mesh. We also note that this increase is highest in the middle cells, i.e., the cells surrounded by cells on two sides in the fiber direction. Along the sheet and normal directions there is much less variation between the different cells.

### Spatial distributions of strain and stress

**Fig. 9:**
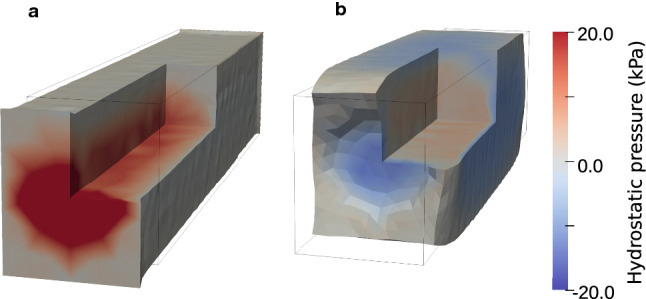
Hydrostatic pressure and displacement distributions. Spatial plots of unknowns ${\textbf{u}}$ and *p* that we solved for; the hydrostatic pressure *p* the colormap plotted on the domain deformed according to the displacement ${\textbf{u}}$ subject to stretching in the fiber direction (**a**) and contraction (**b**)

**Fig. 10:**
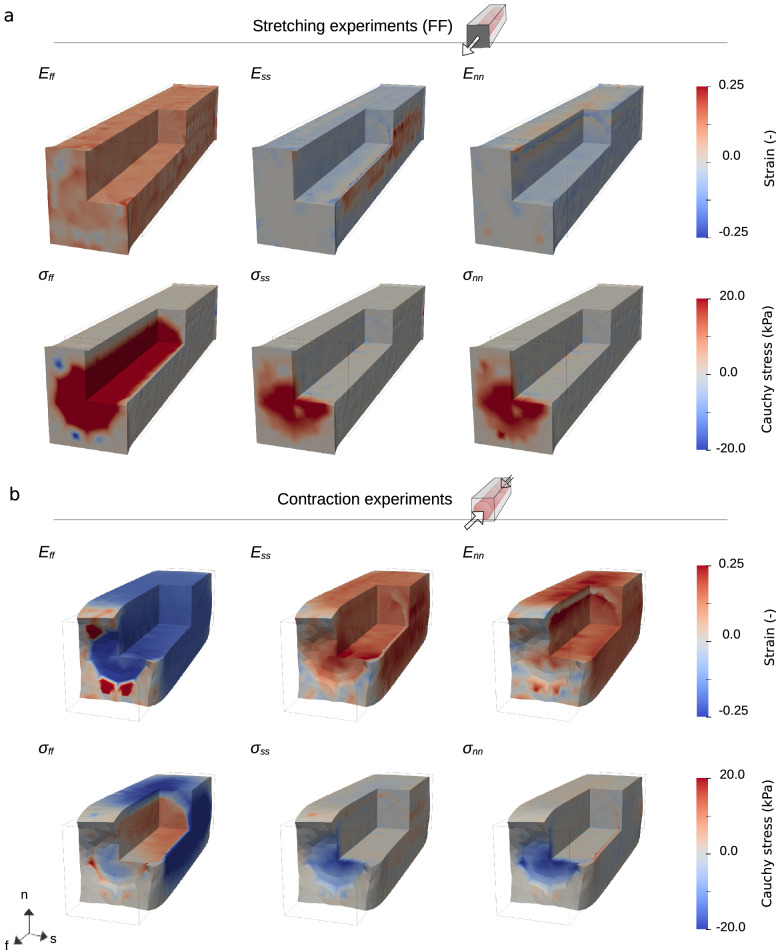
Spatial strain and stress plots for a single cell. Spatial plots of strain and stress subject to stretching in the fiber direction (**a**) and contraction (**b**), for a single cell. We report the normal components of the Green–Lagrange strain tensor ${\textbf{E}}$ (14) and the Cauchy stress tensor $\mathbf {\sigma }$ (15). Some values have magnitudes outside the range displayed—in particular, the $\sigma _{ff}$, reaches about 40 kPa under stretch (in the intracellular space), and about −30 kPa under contraction (in the extracellular space). See also Movie 1 and Movie 3

**Fig. 11:**
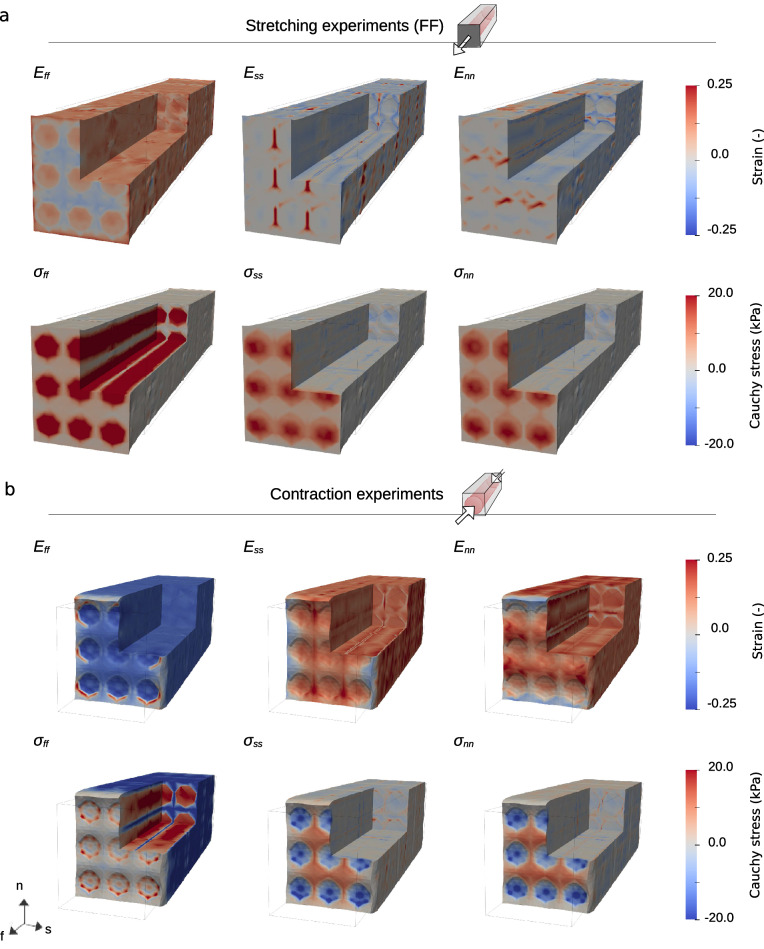
Spatial strain and stress plots for $3 \times 3 \times 3$ cells. Spatial plots of stretching in the fiber direction (**a**), contraction, at peak (**b**), for a mesh consisting of $3 \times 3 \times 3$ cells. As in Fig. 10, we report the normal components of the Green–Lagrange strain tensor ${\textbf{E}}$ (14) and the Cauchy stress tensor $\mathbf {\sigma }$ (15). Values outside the range displayed here reach magnitudes twice as high as for a single cell −80 kPa and −60 kPa, for $\sigma _{ff}$ under fiber direction stretch and under contraction, respectively. See also Movie 2 and Movie 4

**Fig. 12:**
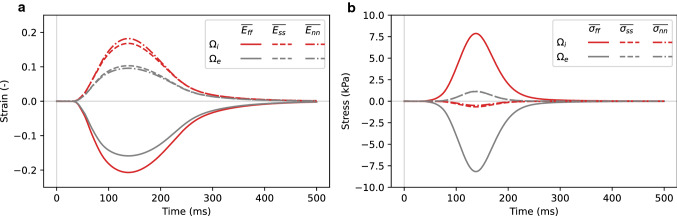
Normal strain (**a**) and stress (**b**) components for each of the two subdomains, considering a single cell under contraction. Normal components of ${\textbf{E}}$ (15) and ${\varvec{\sigma}}$ (14), averaged over the intracellular and extracellular subdomains $\Omega _i$ and $\Omega _e$ – see Equations (16) and (17) – over the first 500 ms of a cardiac cycle

**Fig. 13:**
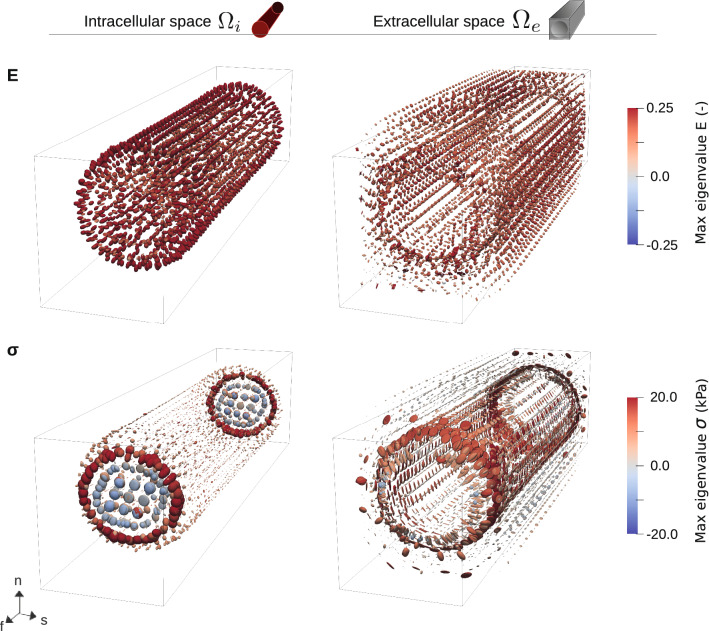
Tensor glyph plots for a single cell under contraction. The top panels display strain, and the bottom panels stress values; for the intracellular subdomain $\Omega _i$ (left) and the intracellular subdomain $\Omega _e$ (right). The tensor values are represented using deformed spheres, inserted at all node points in the mesh. Elongated glyph means we have one dominant principal direction; flat glyphs mean we have two; the spherical ones indicate that all three are equal in magnitude. The size of a glyph corresponds to the magnitude of the eigenvalues at that point, and each glyph is colored according to the largest of these, in magnitude. See also Movie 3 and Movie 4

Spatial distributions of the pressure, strain and stress values for fiber direction stretching and contraction experiments are presented in Figs. 9, 10, 11, and 13. All spatial plots are deformed according to the respective displacement fields. For the contraction experiment for the single cell, we also include plots of strain and stress values averaged over each subdomain in Fig. 12. Movies of the evolution of these quantities under different levels of stretch and over time are presented in the supplementary material, see Movie 1-Movie 4.

For the stretching experiments, the values are plotted at maximum stretch, while for contraction, this is the state in which the cell reaches the highest contraction, corresponding to the peak of the active strain in Fig. 3d. We report the normal components of ${\textbf{E}}$ (14) and $\mathbf {\sigma }$ (15). Note that, following the continuity assumption (3) the normal stresses are continuous across the membrane. For stresses in the other directions, however, we predominantly see a clear discontinuity here.

We note that for both stretching and contraction experiments, the pressure field, as displayed in Fig. 9, contributes to about half of the stress in the fiber direction, consistent in sign and magnitudes. The pressure contribution to the stress values is the same along the diagonal entries (per definition, see Eq. (15)), while being zero for all shear entries. However, we can observe that for most of the domain the total stress distributions in the sheer and normal directions are mostly zero-valued, implying that the stress arising from (6) and the stress from the pressure have opposite signs and mostly cancel each other out.

#### Stretching experiments

As displayed in Fig. 10a and 11a, we can observe fairly uniform strain values everywhere in the domain for the stretching experiments. There is, however, a subtle discontinuity along the membrane for components $E_{ss}$ and $E_{nn}$ (visible inside the quarter removed, to the right and on the top, respectively). The stress values, on the other hand, vary considerably. Considering the fiber direction stretch, even though the $E_{ff}$ values along and inside the cell are almost similar, $\sigma _{ff}$ takes significantly higher values in the intracellular subdomain. For the tiled meshes, the patterns observed mostly generalize what we see for the single-cell experiments. However, for $E_{ss}$, and to some degree for $E_{nn}$, we see that the area between the cells is being stretched considerably in their respective directions—without any corresponding strong response in stress values.

In the movies (Movie 1 and Movie 2), we can see the evolution of strain and stress patterns over different stretching levels. In particular, we see strain patterns develop quite early, while, on the scales plotted, the stress patterns develop later. We also see that the $\sigma _{ff}$ is highest along the middle of the cells. This remains true for all steps, but is outside the scale depicted for the final steps in Fig. 10 and  11.

#### Contraction experiments

In contraction, we observed large variations in all scalar fields ranging from $-$0.3 to 0.3 in strain, and from −80 kPa to 40 kPa in stress values. We see this to some degree in the average traces presented in Fig. 12, with highest variation for stresses represented by $\overline{\sigma _{ff}}$. Here we also observe higher orthotropy for strain values than for stress values—the difference between $\overline{E_{ss}}$ and $\overline{E_{nn}}$ is more prominent than the difference between $\overline{\sigma _{ss}}$ and $\overline{\sigma _{nn}}$.

As expected, and consistent with the average values plotted in Fig. 12, we observe mostly negative $E_{ff}$ and mostly positive $E_{ss}$ and $E_{nn}$ values for both domains. The strain values are fairly similar along and inside the cells. In contrast, $\sigma _{ff}$ has a clear discrete transition across the membrane, taking positive values in $\Omega _i$ and negative values in the extracellular subdomain ($\Omega _e$). For the $\sigma _{ss}$ and $\sigma _{nn}$ components, we have low stress in most of the domain. However, there is a small region along the cell with positive values (barely visible as a red line) and a more prominent area at the end of the cell, which has negative values.

Tensor glyphs displaying strain and stress values for a single cell under contraction are plotted in Fig. 13. For intracellular strain values, the glyphs are elongated close to the membrane tangentially. This implies a more prominent expansion close to the membrane than in the middle, and is also visible for the $E_{ss}$ and the $E_{nn}$ components in Fig. 10—somewhat more prominent for $E_{nn}$ than for $E_{ss}$. For extracellular strain values, all glyphs are spherical and somewhat smaller, indicating a more evenly distributed strain distribution with no prominent principal component.

We observe far more prominent stress values in the extracellular subdomain than in the intracellular subdomain. These are mainly concentrated close to the membrane, also in a tangential pattern. We can observe that these are flat glyphs, indicating two prominent principal components. One of these components coincides with the fiber direction, while the other follows the cell shape circularly. These glyphs, highly prominent in this plot, correspond exactly to the subtle red lines barely visible for components $\sigma _{ss}$ and $\sigma _{ff}$ in Fig. 10. We observe flat glyphs at each end of the cell for the intracellular subdomain, also orientated perpendicular to the fiber direction. In the middle section of the cell, however, we have elongated glyphs oriented in the fiber direction, indicating that the main contribution to all the stresses is in this direction.

We observe general higher strain and stress values for the tiled meshes compared to the single-cell experiment. In particular, we have increased magnitude for $\sigma _{ff}$ values, both for the intracellular and extracellular space. We also see that strain values between the cells changes—here, they obtain negative ($E_{ff}$) or positive ($E_{ss}$, $E_{nn}$) values, following the cells surrounding them.

Both color maps and tensor glyphs are also included in movies for contraction, see Movie 3 and Movie 4. Many of the same observations as outlined above can be done here, but we also see that we start with a prominent strain concentration close to the membrane, while the stress concentration in the extracellular domain emerges later on. We also note that the prominent intracellular $\sigma _{ff}$ component is emerging first and is taking highest values close to the connection between the cells.

### Scalability

The performance and memory consumption for the weak and strong scaling experiments are displayed in Tables 4 and 5. The solver required 43–46 Newton iterations in total for every case.

**Table 4:** Performance and weak scaling for 10 continuation steps (15% stretch in the fiber direction)

Cardiac	Nodes	CPUs	Max DOFs	Time	Parallel	Max memory	Memory
cells			per CPU	[h:mm:ss]	efficiency	per CPU [GB]	efficiency
2	1	1	40 702	0:06:23	${-}$	3.49	${-}$
2 $\times$ 2	2	2	39 760	0:11:06	0.58	4.79	0.73
2 $\times$ 2 $\times$ 2	4	4	38 334	0:15:54	0.40	5.93	0.59
4 $\times$ 2 $\times$ 2	8	8	38 250	0:21:13	0.30	6.54	0.53
4 $\times$ 4 $\times$ 2	16	16	38 718	0:41:42	0.15	8.29	0.42
4 $\times$ 4 $\times$ 4	32	32	37 190	1:22:30	0.08	10.86	0.32
8 $\times$ 4 $\times$ 4	64	64	37 895	2:45:07	0.04	15.64	0.22

**Table 5:** Performance and strong scaling for 10 continuation steps (15% stretch in the fiber direction), using a mesh representing $4 \times 4 \times 4$ cardiac cells

Nodes	CPUs	Max DOFs	Time	Speedup	Parallel	Memory	Memory
		Per CPU	[h:mm:ss]		efficiency	[GB]	efficiency
6	6	193 941	5:53:22	${-}$	${-}$	199.21	${-}$
6	12	97 105	3:42:03	1.59	0.796	230.49	0.864
6	24	51 729	2:17:59	2.56	0.640	277.11	0.719
6	48	25 727	1:40:06	3.53	0.441	360.60	0.552
6	96	12 875	1:02:41	5.64	0.352	474.56	0.420
6	192	6 544	0:58:39	6.03	0.188	688.25	0.289

**Fig. 14:**
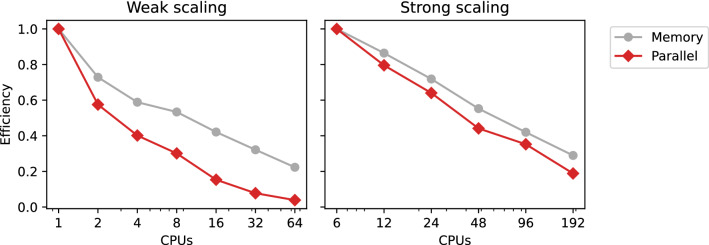
Weak and strong scaling performance. Memory- and parallel (time) efficiency of the weak and strong scaling experiments relative to the baselines of one and six CPUs, respectively. For both experiments, these corresponds to numbers taken from the *Parallel efficiency* and *Memory efficiency* columns in Table 4 and Table 5

The parallel efficiency and memory efficiency for the scaling experiments are shown in Fig. 14. For the weak scaling experiments, we report the baseline time over the measured time for each measurement and the baseline memory divided by the measured memory. For the strong scaling experiments, we report the baseline time, times the number of nodes (6), divided by the measured time and the number of processes, and baseline memory divided by the measured memory.

For the weak scaling experiments, one would ideally hope for the CPU time to remain constant as the problem size and number of CPUs increase proportionally. Unfortunately, this is not the case, and both the CPU time and memory increase significantly as the problem size (number of cardiac cells) increases, despite the work per processor remaining constant. These results indicate that some parts of the direct solver do not scale well.

In the case of the strong scaling, there is clearly some speedup from increasing the number of CPUs, where in particular we observe fairly good scaling of the LU factorization stage. Most of the time is initially spent in this stage, which goes from about 420–480 seconds per Newton iteration when using 1 CPU per node to about 25 seconds per Newton iteration when using 32 CPUs per node. However, the solver must also compute row and column permutations. The row permutation, computed using a serial algorithm, takes about 14 seconds per Newton iteration regardless of how many CPUs are used. The calculation of the column permutation requires more time as the number of CPUs per node increases, taking up to 26 seconds per Newton iteration. Ultimately the computation of row and column permutations limits the scalability of the solver.

## Discussion

We have presented a cardiac model for single cells and small collections of cells, where these cells are embedded in a matrix with differing material properties. It takes into account an explicit geometrical cell configuration that allows for refinement of cardiac tissue mechanics in a more physiologically relevant manner than one can achieve by simply using finer meshes and a homogenized tissue model. This work extends the mechanical aspect of cell-based modeling of single cells (Ruiz-Baier et al. 2014; Gizzi et al. 2015; Garcia-Canadilla et al. 2017; Lenarda et al. 2018; Tracqui et al. 2008) by including the extracellular material. Our results indicate that this inclusion is important for the quantification of intracellular stresses.

### Model parameterization

We used experimental data to parameterize our model, matching six parameters to data from nine different deformation modes, finding a reasonable fit. There is a fairly large variance in the optimal parametrization of each individual sample, which may reflect on variations in the underlying experimental data. Some general patterns observed here is that typically either the $a_e$ or the $a_i$ parameter is high, and the other low. We also see that the fiber direction stiffness parameters $a_{if}$ and $b_{if}$ vary across almost the whole range of allowed parameters, which could arise from varying fiber dispersion in the samples. We also noted that starting the optimization from different starting values did not change the main patterns in the final results. There were certainly some differences indicating local minima, but when averaged out this resulted in no significant differences. As displayed in Fig. 7, the differences between the single cell geometry and the multicellular geometry in computed load during stretching and shearing simulations are minor. This indicates that performing parameter optimizations for passive behavior on a single-cell geometry can, within reason, be extrapolated to multicellular domains.

We were able to capture differences in the stress–strain ratio in different directions transverse to the fiber direction by incorporating geometrical differences, i.e., by making the matrix thicker in the normal direction than in the sheet direction, rather than through additional terms in our strain energy function. The sensitivity analysis of separate intra- and extracellular stresses displays interesting dynamics between the two subdomains. The extracellular material parameters, $a_e$ and $b_e$, proved to be most important for intracellular stresses. Considering the load values, based on the relative sensitivity, we see that both stretching and shearing experiments are important to perform and use in the parametrization to capture these two accurately. The sensitivity analysis also displays that across the stress values, the first order and total Sobol indices are fairly similar, indicating little interaction between the different parameters. For the load values we observed a larger difference between the first order and total indices, indicating a higher degree of interaction.

The formulation used for the intracellular strain energy function $\psi _i$ (9) is very similar to the one used in other works for isolated cardiomyocytes (Tracqui and Ohayon 2009; Ruiz-Baier et al. 2014; Gizzi et al. 2015), and can physiologically be motivated by the mechanical contributions of the sarcomere structure found within the cells. Other models for cardiomyocytes (Tracqui et al. 2008; Okada et al. 2005; Garcia-Canadilla et al. 2017; Lenarda et al. 2018) have assumed isotropy in the intracellular domain, not assuming that there is any difference between stress–strain values in different directions. This could, in particular, be a good assumption for cells and cell collections developed *in vitro*, which as immature cells have a less developed sarcomere structure. For the extracellular strain energy function $\psi _e$ (10) it could in particular be relevant to compare with Sharafi and Blemker (2011) and Zhang and Gao (2012). In Sharafi and Blemker (2011), a term enhancing the shear stiffness is included, while in Zhang and Gao (2012) the authors utilize an isotropic formulation, modeling both subdomains as Mooney-Rivlin materials. Inspired by the first one, we explored several formulations including additional shear components, as well as formulations with additional transverse stiffness. In all cases, however, these were found to be redundant—our optimization script found the corresponding parameters to be zero across most of the samples. The difference in norm found by the optimization script remained fairly similar with or without these extra terms, also for the non-zero cases. As such, we believe the dynamics captured with these potential extra terms is already captured in the current formulation. Further studies are, however, needed to better determine the most representative constitutive relationships in this model, for each subdomain.

Compared to the study in Gizzi et al. (2015), our parameters $a_i$, $b_i$, $a_{if}$ and $b_{if}$ are all stiffer. They considered a single cell, employing a similar model to the one used for the intracellular space. Data are obtained by curve fitting based on the polynomial strain energy function used in Tracqui and Ohayon (2009), which again is based on data published in other studies. Compared to the original study in Kakaletsis et al. (2021), from which we have the experimental data (for tissue samples), we observe an increase in the anisotropy for the intracellular space. We found parameters $a_{if}$ and $b_{if}$ to take values 19.83 kPa and 24.72, respectively. These are comparable to the parameters $a_f$ and $b_f$ as listed in the original paper, in their parameterization of a corresponding homogenized tissue-level model. Across their four strategies, the $a_f$ parameters ranged between 3.85 and 6.60 (kPa), and $b_f$ between 4.34 and 10.79. We see that the intracellular space has a significant increase in degree of anisotropy compared to the homogenized tissue-level model. This can be explained by the isotropic assumption for the extracellular space—to match tissue-level experiments with decreasing anisotropy in one domain, one needs to increase the anisotropy in the other.

The main limitation of the parameterization process might be that, although the model itself is designed for taking into account explicit cellular geometries, we are using an idealized one. In particular, we have a much larger volume ratio between the intracellular and extracellular space than realistic. Our intracellular to extracellular ratio is almost 1 to 1, while values in the literature report the volume ratio to be around 4 to 1 (Olivetti et al. 1991). This is likely to lead to an overestimation of $a_{if}$ and $b_{if}$, as the stiffness is defined over a volume which is too small. In addition, we have added extra padding between every sheet of single cells. In reality there should be a few cells per sheet, surrounded by perimysium layers on both sides, which will have an impact differences between the sheet and normal directions. We also only connect the cells in the fiber direction, in an artificially regular manner. As displayed in Fig. 8, there is a huge degree of interaction between the cells in this direction and only marginal interaction in the other directions, tracking stress values under contraction. Connecting them in the sheet direction as well, which is, e.g., done in the electrophysiological model in Tveito et al. (2017), would most likely bring in more interaction in this direction as well. Capturing all of these closer-to-realistic aspects would be of major interest, bringing us closer to capturing real physiological features, but would also require us to use far more advanced meshing techniques.

Histological information is included in the data published (Kakaletsis et al. 2020). One could potentially make geometries based on these images, which would most likely lead to a significant improvement of the fit and new exciting results. The main problem here, however, is the size of the tissue samples. All of the samples were cut to have approximate dimensions $10 \text { mm} \times 10 \text { mm} \times 10 \text { mm}$, for which one can estimate a couple of millions of cells, far more than the 128 cells we are, at max, considering in our study. An additional limitation of our optimization procedure is that we only consider the positive values, while the original data also included negative ones, i.e., compressive stress and shear in two directions. Including both sides could potentially lead to a better parameterization—in particular, because we have a conditional term in the fiber direction, dependent on whether we have stretching or compression. We also assumed continuity of stresses across the membrane (3), implying that the membrane has no stiffness. Incorporating some stiffness for the membrane would probably be more realistic and likely to affect the cell–matrix dynamics observed. It could potentially also give a better fit to the experimental data. For simplicity, however, we chose not to include it.

As described above, a study using a cell-based model, paired with stretch and image-based geometries and corresponding stretch/strain experiments on a smaller tissue sample would probably be the most rigorous and accurate way for proper separate characterizations of material properties of the cardiac cells and the matrix. It could also make sense to explore geometries generated artificially, capturing more realistic features, e.g., by allowing for a non-regular tiling pattern or matching the extracellular/intracellular volume ratio. Finally, it would be interesting to compare how the parametrization of our model would change when parametrized based on data from other experimental studies.

### Toward a more physiologically relevant model

The results presented in this paper are highly dependent on the underlying geometrical cell configuration. The strain and stress plots display spatial patterns, following the cell geometries, with clear transitions along the membrane. Under contraction, this is, in particular, visible as a clear tangential-normal pattern. Here stress values in the extracellular domain are concentrated in directions tangential to the membrane, perpendicular to prominent intracellular strain concentrations, oriented normal to the cell membrane. Through differences in the geometry in the sheet and normal direction, we were able to capture a fully orthogonal behavior of the tissue. This applies both for stretching and shearing experiments, as displayed in Fig. 4, as well as for stress and especially strain values under contraction, as displayed in Fig. 12.

Real geometries are much more complicated—for a single cell, the domain has a more complex shape, and for multiple cells, the cells self-organize in more complex, non-regular patterns. Images of cells can be used to construct more realistic geometries, as done in, e.g., Ruiz-Baier et al. (2014); Gizzi et al. (2015); Garcia-Canadilla et al. (2017); Tracqui et al. (2008) considering single cells, and to some degree in in Sharafi and Blemker (2011), considering a cross-section through nine cells. For the latter study, force values reported are within a comparable range to stresses observed in our study; however their setup is quite different from ours, making it hard to compare these directly.

Most of the models mentioned above include calcium dynamics, with experimental spatial-temporal calcium measurements driving the model. A limitation of our model is that it does not couple strain dependencies back to the active tension, which would be more physiologically correct. In particular it would be of interest to include this for comparison to the work performed by Tracqui et al. (2008). This was designed to match the experimental setup explored in Qin et al. (2007), and explains key sub-processes in cell–material dynamics subject to increasing stiffness of the surrounding material. A fully coupled model would, however, only matter for capturing active contraction dynamics, while the stretching and shearing experiments determining the material parameters would remain unaffected.

The proposed model has the ability to represent micro-scale heterogeneities in intracellular and extracellular matrix properties, e.g., by allowing the individual cells to have different properties. Given an image-based geometry of actual cardiac cells, far more realistic simulations could potentially be achieved. Extending our purely mechanical model to take into account calcium and sarcomere length dynamics would also bring in intracellular heterogeneities for the active tension, with resulting new strain and stress patterns. Measurements of subcellular mechanical structures and calcium measurements, as explored in, e.g., Reichardt et al. (2020); Garcia-Canadilla et al. (2017); Blatter et al. (2003); Deckx et al. (2019), could be used within such a framework for exploration of heterogeneities within a single cell or a small collection of a few cells. A key focus here could, for example, be to differentiate between heterogeneities in material properties versus heterogeneities in the activation parameters. An alternative approach, focusing on cell–cell and cell–matrix interactions, would be to keep the mechanical properties and calcium levels homogeneous within each cell, and instead utilize cell-based calcium measurements, as reported, e.g., in Jones et al. (2018). The numerical framework, as developed for computational resources presently available to us, is currently far from capable of capturing the whole heart. It might, however, be possible to use it either to capture the mechanics of *in vitro* cell collections, such as cardiac microtissues (Zhang et al. 2015), or to examine in detail small sections of the myocardium, to understand how local interactions may propagate up to tissue and organ levels.

A particular area of interest for the model is in disease modeling, targeting diseases affecting mechanics on this scale. Our model could, for example, be used to study the impact of hypertrophy, characterized by changes in the cell geometries (Göktepe et al. 2010). By explicitly modeling the intracellular space, one could look at changes to the myocyte during eccentric or concentric remodeling by altering the length and diameter of the cells. This could be useful in helping to delineate how mechanical triggers such as stress and strain drive these remodeling processes, by more accurately determining the stress and strain the myocyte experiences. Here it would be interesting to understand how remodeling-based changes to the cell geometries can normalize altered load or contribute to continued remodeling stimuli. Another application could be in modeling scarring, in which the cell structures in the damaged part change upon healing (Rog-Zielinska et al. 2016). In the scarred regions, fibroblast cells differentiate to myofibroblast, which have contractile properties (Baum and Duffy 2011). In principle, there is no reason our cell-based model should only work for cardiomyocytes; it would be fairly straightforward to extend the geometrical framework to including different cell types with different mechanical and contractile properties.

Hypertrophy and scarring are both common causes of cardiac fibrosis (Maulik and Mishra 2015; Hinderer and Schenke-Layland 2019), which also can be represented more explicitly in a cell-based framework than in traditional tissue-level models. This includes interstitial fibrosis, in which the matrix stiffness increases, or replacement fibrosis, in which cells are replaced by a collagen network (Hinderer and Schenke-Layland 2019). In our model, we could capture this by, respectively, increasing the extracellular material parameters and by replacing certain cells with more matrix in the geometry. For the first one, in particular, we have demonstrated that the cardiomyocyte stresses are highly dependent on the matrix stiffness, so even a small increase in matrix stiffness could be expected to have a large impact. Modeling this explicitly on a cell-based level could provide physiological insights and understanding of how these diseases work on the relevant scale. In a long-term perspective, following the development of more efficient simulations, our modeling framework may be well-suited to represent larger tissue samples of clinically relevant sizes. Such an explicit representation could, for example, be used as an alternative to the more common statistical representations of fiber dispersion.

### Implications of continuity and discontinuity assumptions at the membrane

In our implementation of the model, we solved for displacement ${\textbf{u}}$ and pressure *p* in a Taylor-Hood discretization space, assuming continuity of both in the whole domain. Furthermore, we took $\psi$ (6) to be discontinuous across the membrane, implying that the first Piola-Kirchhoff stress tensor can be discontinuous as well—except from normal to the membrane, where we assumed continuity (3). We also assumed incompressibility in both subdomains.

Our approach is very similar to the one considered in Tracqui et al. (2008), in which three subdomains are considered (the substrate, the cell, and the nucleus), and the whole system is solved simultaneously. As in our case, they assume continuity of displacement and stresses across the membranes—but in all directions. In Farsad and Vernerey (2012), on the other hand, continuity is neither assumed for displacement nor stresses. They also use the extended finite element method (XFEM) rather than FEM. Here, the discontinuities at the membrane are represented by Heaviside functions instead of explicitly representing the intersection in the mesh. They observe stress concentrations close to the membrane, consistent with our results. Yet another alternative could be to employ a discontinuous Galerkin (DG) method, as outlined, e.g., in Ten Eyck et al. (2008). We also note that in the electrophysiology cell-based model in Tveito et al. (2017), they employ a splitting scheme in which the equations for the intracellular space, the membrane, and the extracellular space are solved separately. We note that an incompressible formulation is used in Ruiz-Baier et al. (2014); Lenarda et al. (2018) for isolated myocytes, and a nearly incompressible formulation is used in Zhang and Gao (2012) for skeletal cells, surrounded by an endomysium layer, for both subdomains.

In our case, we found that the base mesh combined with the Taylor-Hood discretization remains a reasonable choice. Using a continuous function space for the pressure is, in particular, a limitation of our work, as there is no physical reason that neither the pressure nor the pressure-dependent stresses should be continuous. As displayed in Fig. 9, the pressure fields are somewhat blurred out across the membrane—but this appears to be a fairly minor artifact. XFEM and DG methods could be used to capture the discontinuity, however, they include a more complex mathematical framework and are more expensive discretizations, which do not seem necessary for our work. A splitting scheme could potentially also be developed for the mechanical model—however, again, this would make the methods more complex without necessarily being more precise at this stage. Cardiac tissue is known to be compressible (Yin et al. 1996; Nolan and McGarry 2016), in which the cells, which primarily consist of water, are close to incompressible, while the matrix’ volume changes significantly under pressure. The matrix is estimated to be 100–1000 times more compressible than the cells it surrounds (Dolega et al. 2021). Alternatively one could use a nearly incompressible formulation, as done in Telle et al. (2021), but with a much higher penalty parameter for the intracellular subdomain. High penalty parameters are, however, associated with locking (Hadjicharalambous et al. 2014; Karabelas et al. 2022), which both can lead to numerical instabilities and underestimation of variables of interest. For these reasons we chose an incompressible formulation. This challenge could, however, be overcome using other kinds of elements, as widely explored in (Karabelas et al. 2022).

A more rigorous comparison between different numerical schemes would probably lead to the development of more efficient and accurate methods. In particular, if one wants to couple the mechanics with the underlying electrophysiology, the splitting scheme could be more appropriate. Alternative formulations for either incompressible or nearly incompressible formulations would be prudent to explore in the future, and it would in particular be interesting to see the impact of defining these differently in each subdomain.

### HPC considerations

Through our scaling experiments, we explored limitations of the presented numerical solution approach both with respect to time and memory consumption. We observe reasonable speedup for some parts of the direct solver, such as for the LU factorization stage. However, other parts become increasingly costly, such as the computation of row and column permutations, which are needed for robustness and to reduce fill-in of the computed factorization. As the problem size increases, these parts seem to dominate the execution time.

As considered in, e.g., Whiteley (2017); Brune et al. (2015), iterative methods combined with suitable preconditioners might give better performance for nonlinear elasticity problems. In terms of problem size, our largest meshes lie somewhere in between the second largest and largest problem size considered in Whiteley (2017), indicating that our problem is comparable to their experiments in terms of degrees of freedom. Operator splitting schemes have proven to give significant speedup for the electrophysiological EMI model, as considered in Jæger et al. (2021), and similar approaches would be interesting to investigate from a mechanical perspective as well. Hexahedral meshes have been demonstrated to be more accurate than tetrahedral meshes, which we have used, as reported in, e.g., Karabelas et al. (2022); Oliveira and Sundnes (2016) for related problems. From an efficiency perspective, this implies one could get better solutions with coarser meshes, which could be worth considering for future work.

Limited scalability obviously affects the generalization of our simulations—using our framework, we can only consider small collections of cells. Implementing preconditioners and operator splitting schemes are, however, not always straightforward and can be considered a separate extensive research question.

In the future, it would be prudent to investigate alternative solvers. It would also be interesting to see whether changing, e.g., the mesh structure would lead to different numerical properties, both with respect to convergence and with respect to efficiency. Physiologically relevant extensions of the model presented in this paper—e.g., image-based geometries, coupling to electrophysiological models, utilizing experimentally measured calcium cell-wise or high-resolution single-cell experiments—are dependent on high-resolution meshes. Anyone investigating such questions would presumably meet similar scalability limitations. Pushing these limits would enable investigation of larger problems, opening up for investigating a wide range of new research questions.

## Conclusions

We have introduced a cell-based framework for modeling cardiac tissue, parametrized based on experimental data. This was done by pairing stretching and shearing experiments performed on cardiac tissue samples with corresponding virtual experiments. We observe that the adaption to a geometrical framework, based on relatively simple extensions of existing ideas used for tissue level models, give rise to striking spatial strain and stress values patterns and opens up for numerous new interesting research questions. Utilizing our model, we have been able to differentiate between deformation and stresses in the cells from the matrix surrounding them. Moving to multicellular and finer meshes have, however, proven computationally expensive, as demonstrated by our scaling and convergence experiments.

Our work demonstrates that it is feasible to work with a discretized model which explicitly represents the cells, ranging from a single cell to small collections of cells. Our geometrical approach can further be extended by using more realistic geometries or be coupled with calcium dynamics, which could be used for new studies leading to an improved understanding of cardiac mechanics.

## Supporting information

We include movies for the spatial distributions, considering fiber direction stretch and contraction. For all movies we display normal components of and tensor glyphs for the Green–Lagrange strain tensor ${\textbf{E}}$ (14) (top row) and the Cauchy stress tensor ${\sigma }$ (15) (bottom row).

*Movie 1*

Fiber direction stretch (FF), single cell. Strain and stress components for a single cell subject to stretching in the fiber direction. Maximum stretching state values, at 10% stretch, are also displayed in Fig. 10.

*Movie 2*

Fiber direction stretch (FF), tiled cells. Strain and stress components for a $3 \times 3 \times 3$ cells subject to stretching in the fiber direction. Maximum stretching state values, at 10% stretch, are also displayed in Fig. 11.

*Movie 3*

Contracting cell, single cell. Strain and stress components for a single contracting cell under contraction, for half a cardiac cycle. Maximum contracted state values are also displayed in Fig. 10 and Fig. 13.

*Movie 4*

Contracting cells, tiled cells. Strain and stress components for $3 \times 3 \times 3$ cardiac cells under contraction, for half a cardiac cycle. Maximum contracted state values are also displayed in Fig. 11.

### Supplementary Information

Below is the link to the electronic supplementary material.

Supplementary file 1 (mp4 10709 KB)

Supplementary file 2 (mp4 11372 KB)

Supplementary file 3 (mp4 6581 KB)

Supplementary file 4 (mp4 6908 KB)

## Appendix: Weak form and boundary conditions

In this appendix, we provide a more rigorous mathematical framework for how the weak form was derived, and how the different boundary conditions for the different deformation modes were imposed. All deformation modes are sketched and explained in Fig. 3.

### Derivation of the weak form

Equilibrium of stresses is, in this paper, expressed using the first Piola-Kirchhoff stress tensor, as given in Eq (1). In this work, we employed an active strain formulation and assumed incompressibility imposed by a Lagrangian multiplier. Following the derivations in Ambrosi and Pezzuto (2012) and Holzapfel (2000), we can incorporate these changes in the first Piola-Kirchhoff stress tensor, which then reads

19 $$\begin{aligned} {\textbf{P}} =  \textrm{det} (\mathbf {F_a}) \frac{\partial \psi (\mathbf {F_p})}{\partial \mathbf {F_p}} \mathbf {F_a}^{\mathrm {-T}} + J p {\textbf{F}}^{-\textrm{T}} \end{aligned}$$

where an equilibrium solution still is given by (1). ${\textbf{P}}$ is dependent on the displacement ${\textbf{u}}$ and the hydrostatic pressure *p*, which represent the unknowns.

One can define appropriate function spaces *V* and *Q*, defined over $\Omega$, for the displacement and hydrostatic pressure respectively. Starting with the strong form of our problem (1), one can consider test functions ${\textbf{v}} \in V$ and take the integral over $\Omega _i$ and $\Omega _e$ separately:

20 $$\begin{aligned} \int _{\Omega _i} (\nabla \cdot {\textbf{P}}) \cdot {\textbf{v}} \textrm{d}{\textbf{X}} = 0 \end{aligned}$$

and

21 $$\begin{aligned} \int _{\Omega _e} (\nabla \cdot {\textbf{P}}) \cdot {\textbf{v}} \textrm{d}{\textbf{X}} = 0 \end{aligned}$$

then perform integration by parts in order to obtain

22 $$\begin{aligned} \int _{\Omega _i} {\textbf{P}}: \nabla {\textbf{v}} \textrm{d}{\textbf{X}} =  \int _{\Gamma } (\mathbf {n_i} \cdot {\textbf{P}}) \cdot {\textbf{v}} \textrm{ds}  \end{aligned}$$

23 $$\begin{aligned}{} & {}  \int _{\Omega _e} {\textbf{P}}: \nabla {\textbf{v}} \textrm{d}{\textbf{X}} =  \int _{\Gamma } (\mathbf {n_e} \cdot {\textbf{P}}) \cdot {\textbf{v}} \textrm{ds}+ \int _{\partial \Omega } (\mathbf {n_e} \cdot {\textbf{P}}) \cdot {\textbf{v}} \textrm{ds}. \end{aligned}$$

We assume the continuity of stresses across $\Gamma$ as given in (3), which implies that the membrane has no stiffness. If one wanted to incorporate some stiffness here, which physically probably would be meaningful, this could be an equation to change. For simplicity, though, we will keep the formulation as it is, implying that

24 $$\begin{aligned} \int _{\Gamma } (\mathbf {n_i} {\textbf{P}}) \cdot {\textbf{v}} \textrm{ds} = - \int _{\Gamma } (\mathbf {n_e} {\textbf{P}}) \cdot {\textbf{v}} \textrm{ds} \end{aligned}$$

By adding (22) and (23), the integral terms on $\Gamma$ cancel, and we can combine the integrals to a integral over all of $\Omega$,

25 $$\begin{aligned} \int _{\Omega } {\textbf{P}}: \nabla {\textbf{v}} \textrm{d}{\textbf{X}} - \int _{\partial \Omega } (\mathbf {n_e} {\textbf{P}}) \cdot {\textbf{v}} \textrm{ds} \,= \,& 0. \end{aligned}$$

On the outer boundary $\partial \Omega$, we apply either Dirichlet boundary conditions or zero Neumann conditions, which makes the surface integral over $\partial \Omega$ vanish.

For the incompressibility condition, given by Eq (5), we can multiply the equation with a test function $q \in Q$ to obtain

26 $$\begin{aligned} \int _{\Omega } (J - 1) q \textrm{d}{\textbf{X}} = \, 0 \end{aligned}$$

where no decomposition of the subdomain is needed.

By adding these two equations, our weak form becomes

27 $$\begin{aligned} \int _{\Omega } {\textbf{P}} ({\textbf{u}}, p): \nabla {\textbf{v}} + q(J ({\textbf{u}}) - 1) \textrm{d}{\textbf{X}} =\, 0 \end{aligned}$$

and our problem can be formulated as follows: Find a solution $({\textbf{u}}, p)$ such that the above equation holds for all ${\textbf{v}} \in V$ and all $q \in Q$.

The above equation is solved as a stationary problem, in which time dependent variables are kept constant. These variables either define the boundary displacement, for stretching/shearing experiments, or the active tension (5), for contraction experiments.

### Boundary conditions for stretching experiments

We imposed Dirichlet boundary conditions on two of the surfaces, step-wise increasing one of the components, giving a proportional stretch of $\lambda$ of the whole domain. If, e.g., if $\lambda$ is increased up to 0.15 for the fiber direction stretch (FF), the deformed domain becomes 15% longer in this direction.

The remaining components on these two surfaces were fixed to zero, while we allowed free movement on the other surfaces. Combined with the incompressibility constraint, this implies a shortening in the direction perpendicular to the stretching direction.

Mathematically the boundary conditions described above can be expressed as

28 $$\begin{aligned} {\textbf{u}} = (0, 0, 0) \quad \text {on } S_1 \end{aligned}$$

29 $$\begin{aligned} {\textbf{u}} = (\lambda , 0, 0)\quad \text {on } S_2 \end{aligned}$$

for the fiber direction stretch. Similarly, for the sheet direction stretch, we have

30 $$\begin{aligned} {\textbf{u}} = (0, 0, 0)\,\,\text {on } S_3 \end{aligned}$$

31 $$\begin{aligned} {\textbf{u}} = (0, \lambda , 0)\,\quad\text {on } S_4 \end{aligned}$$

and for the normal direction stretch, we have

32 $$\begin{aligned} {\textbf{u}} = (0, 0, 0)\,\quad\text {on } S_5 \end{aligned}$$

33 $$\begin{aligned} {\textbf{u}} = (0, 0, \lambda )\,\quad\text {on } S_6 \end{aligned}$$

For all the surfaces not mentioned above, for each experiment, we impose vanishing Neumann boundary conditions, i.e.,

34 $$\begin{aligned} \mathbf {n_e} {\textbf{P}} = (0, 0, 0). \end{aligned}$$

### Boundary conditions for shear experiments

Similarly to the stretching experiments, shear experiments were performed by imposing Dirichlet boundary conditions on two of the surfaces. One of the components was then increased, giving a proportional shear deformation of $\kappa$. If, e.g., $\kappa$ was increased up to 0.15, the surface on which we apply non-zero Dirichlet boundary conditions is moved a distance corresponding to 15% of the distance between the two surfaces being fixed.

Mathematically, we have, for the FS experiment

35 $$\begin{aligned} {\textbf{u}} = (0, 0, 0) \,\quad \text {on } S_1 \end{aligned}$$

36 $$\begin{aligned} {\textbf{u}} = (0, \kappa , 0)\,\quad\text {on } S_2 \end{aligned}$$

for the FN experiment

37 $$\begin{aligned} {\textbf{u}} = (0, 0, 0)\,\quad\text {on } S_1 \end{aligned}$$

38 $$\begin{aligned} {\textbf{u}} = (0, 0, \kappa )\,\quad\text {on } S_2 \end{aligned}$$

for the SF experiment

39 $$\begin{aligned} {\textbf{u}} = (0, 0, 0)\,\quad\text {on } S_3 \end{aligned}$$

40 $$\begin{aligned} {\textbf{u}} = (\kappa , 0, 0)\,\quad\text {on } S_4 \end{aligned}$$

for the SN experiment

41 $$\begin{aligned} {\textbf{u}} = (0, 0, 0)\,\quad\text {on } S_3 \end{aligned}$$

42 $$\begin{aligned} {\textbf{u}} = (0, 0, \kappa )\,\quad\text {on } S_4 \end{aligned}$$

for the NF experiment

43 $$\begin{aligned} {\textbf{u}} = (0, 0, 0)\,\quad\text {on } S_5 \end{aligned}$$

44 $$\begin{aligned} {\textbf{u}} = (\kappa , 0, 0)\,\quad\text {on } S_6 \end{aligned}$$

and, finally, for the NS experiment

45 $$\begin{aligned} {\textbf{u}} = (0, 0, 0)\,\quad\text {on } S_5 \end{aligned}$$

46 $$\begin{aligned} {\textbf{u}} = (0, \kappa , 0)\,\quad\text {on } S_6. \end{aligned}$$

For all the surfaces not mentioned above, for each experiment, we impose vanishing Neumann boundary conditions, i.e.,

47 $$\begin{aligned} \mathbf {n_e} {\textbf{P}} = (0, 0, 0). \end{aligned}$$

### Boundary conditions for active contraction

For the active contraction experiments, the traction along every surface was set to 0:

48 $$\begin{aligned} {\textbf{P}} {\textbf{n}}_e = 0 \qquad \text { on } \partial \Omega . \end{aligned}$$

Due to the Neumann boundary conditions, this formulation does not possess a unique solution. In fact, the formulation has a six-dimensional kernel consisting of rigid deformations, i.e., translation and rotation in a three-dimensional space. We remove these modes by enforcing orthogonality of the solution with respect to the kernel by means of Lagrangian multipliers, as done in, e.g., Kuchta et al. (2016): Let *R* be the space of rigid motions. Using the finite element formulation, we then seek to find displacement ${\textbf{u}}$, pressure *p* and rigid motion ${\textbf{r}} \in R$ such that

49 $$\begin{aligned} \int _{\Omega } ({\textbf{P}}: \nabla {\textbf{v}} + q(J - 1) + {\textbf{r}} \cdot {\textbf{v}}) d{\textbf{x}} =  0 \end{aligned}$$

and

50 $$\begin{aligned} \int _{\Omega } {\textbf{u}} \cdot {\textbf{s}} d {\textbf{x}} =  0\, \end{aligned}$$

for all ${\textbf{v}} \in V$, all $q \in Q$ and all ${\textbf{s}} \in R$.

Here *r* can be interpreted as a Lagrange multiplier enforcing the constraint that the solution ${\textbf{u}}$ is free of translation or rotation with respect to the original configuration. Equation (50) implies that ${\textbf{u}}$ is orthogonal to *R*. In the practical implementation we fix a chosen set of basis vectors, $\left\{ \mathbf {s_i}\right\} _{i=1}^6$, of the six-dimensional rigid motion space *R* and obtain ${\textbf{r}}$ through its expansion coefficients in the basis, i.e., ${\textbf{r}}=\sum _i c_i \mathbf {s_i}$. Here the requirement (50) is equivalent to imposing $\int _{\Omega } {\textbf{u}} \cdot \mathbf {s_i} \textrm{d}{\textbf{X}} = 0$ for all $1\le i\le 6$.

## Declarations

### Code availability

All code developed for this paper has been made publicly available, see Telle (2022).

## References

[CR1] Abhilash A, Baker BM, Trappmann B, Chen CS, Shenoy VB (2014). Remodeling of fibrous extracellular matrices by contractile cells: predictions from discrete fiber network simulations. Biophys J.

[CR2] Alnæs M, Blechta J, Hake J, Johansson A, Kehlet B, Logg A, $$\ldots$$ Wells GN (2015) The FEniCS project version 1 5. Archive of Numerical Software 10.11588/ans.2015.100.20553

[CR3] Ambrosi D, Pezzuto S (2012). Active stress vs active strain in mechanobiology: constitutive issues. J Elast.

[CR4] Avazmohammadi R, Soares JS, Li DS, Raut SS, Gorman RC, Sacks MS (2019). A contemporary look at biomechanical models of myocardium. Annu Rev Biomed Eng.

[CR5] Azad A, Buluç A, Li XS, Wang X, Langguth J (2020). A distributed-memory algorithm for computing a heavy-weight perfect matching on bipartite graphs. SIAM J Sci Comput.

[CR6] Azeloglu EU, Costa KD (2010). Cross-bridge cycling gives rise to spatiotemporal heterogeneity of dynamic subcellular mechanics in cardiac myocytes probed with atomic force microscopy. Am J Phys Heart Circ Physiol.

[CR7] Baum J, Duffy HS (2011) Fibroblasts and myofibroblasts: what are we talking about? J Cardiovascular Pharmacol 574376 10.1097/FJC.0b013e3182116e39 PMC3077448 21297493

[CR8] Bensley JG, De Matteo R, Harding R, Black MJ (2016). Three-dimensional direct measurement of cardiomyocyte volume, nuclearity, and ploidy in thick histological sections. Sci Rep.

[CR9] Blatter LA, Kockskämper J, Sheehan KA, Zima AV, Hüser J, Lipsius SL (2003). Local calcium gradients during excitation-contraction coupling and alternans in atrial myocytes. J Physiol (Lond).

[CR10] Borbély A, Van Der Velden J, Papp Z, Bronzwaer JG, Edes I, Stienen GJ, Paulus WJ (2005) Cardiomyocyte stiffness in diastolic heart failure. Circulation1116774–781. 10.1161/01.CIR.0000155257.33485.6D 10.1161/01.CIR.0000155257.33485.6D 15699264

[CR11] Brune PR, Knepley MG, Smith BF, Tu X (2015). Composing scalable nonlinear algebraic solvers. SIAM Rev.

[CR12] Costa KD, Takayama Y, McCulloch AD, Covell JW (1999). Laminar fiber architecture and three-dimensional systolic mechanics in canine ventricular myocardium. Am J Physiol Heart Circ Physiol.

[CR13] Deckx S, Johnson DM, Rienks M, Carai P, Van Deel E, der Velden Van J, Papageorgiou A-P (2019). Extracellular sparc increases cardiomyocyte contraction during health and disease. PLoS One.

[CR14] Dolega ME, Monnier S, Brunel B, Joanny,J-F, Recho P, Cappello G (2021) Extracellular matrix in multicellular aggregates acts as a pressure sensor controlling cell proliferation and motility. Elife p. 10e63258. (Publisher: eLife Sciences Publications Limited) 10.7554/eLife.63258 PMC8064752 33704063

[CR15] Dongarra JJ, Duff IS, Sorensen DC, Van der Vorst HA (1998). Numerical linear algebra for high-performance computers. SIAM.

[CR16] Farsad M, Vernerey F (2012). An XFEM-based numerical strategy to model mechanical interactions between biological cells and a deformable substrate. Int J Num Methods Eng.

[CR17] Finsberg H, Balaban G, Ross S, Håland TF, Odland HH, Sundnes J, Wall S (2018). Estimating cardiac contraction through high resolution data assimilation of a personalized mechanical model. J Comput Sci.

[CR18] Fomovsky GM, Thomopoulos S, Holmes JW (2010). Contribution of extracellular matrix to the mechanical properties of the heart. J Mol Cell Cardiol.

[CR19] Garcia-Canadilla P, Rodriguez J, Palazzi M, Gonzalez-Tendero A, Schönleitner P, Baličević V, Bijnens B (2017). A two dimensional electromechanical model of a cardiomyocyte to assess intra-cellular regional mechanical heterogeneities. PLOS ONE.

[CR20] Geuzaine C, Remacle J-F (2009). Gmsh: A 3-D finite element mesh generator with built-in pre- and post-processing facilities. Int J Num Methods Eng.

[CR21] Gizzi A, Ruiz-Baier R, Rossi S, Laadhari A, Cherubini C, Filippi S (2015) A three-dimensional continuum model of active contraction in single cardiomyocytes. Modeling the heart and the circulatory system (pp. 157–176). Cham:Springer International Publishing. 10.1007/978-3-319-05230-4_6

[CR22] Göktepe S, Abilez OJ, Parker KK , Kuhl E (2010) A multiscale model for eccentric and concentric cardiac growth through sarcomerogenesis. J Theoretical Biol pp. 2653433–442 10.1016/j.jtbi.2010.04.023 20447409

[CR23] Guccione JM, McCulloch AD, Waldman LK (1991). Passive material properties of intact ventricular myocardium determined from a cylindrical model. J Biomech Eng.

[CR24] Hadjicharalambous M, Lee J, Smith NP, Nordsletten DA (2014) A displacement-based finite element formulation for incompressible and nearly-incompressible cardiac mechanics. Computer Methods in Applied Mechanics and Engineering pp. 274213-236. Elsevier, Publisher 10.1016/j.cma.2014.02.009 PMC4026127 25187672

[CR25] Herman J, Usher W (2017) SALib An open-source python library for sensitivity analysis. J Open Source Softw. 10.21105/joss.00097

[CR26] Hinderer S, Schenke-Layland K (2019) Cardiac fibrosis–A short review of causes and therapeutic strategies. Adv Drug Delivery Rev pp. 14677–82 10.1016/j.addr.2019.05.011 31158407

[CR27] Hogues H, Leon L, Roberge F (1992). A model study of electric field interactions between cardiac myocytes. IEEE Trans Biomed Eng doi.

[CR28] Holzapfel AG (2000) Nonlinear solid mechanics. ics. Sussex:John Wiley & Sons, LTD

[CR29] Holzapfel GA, Ogden RW (2009). Constitutive modelling of passive myocardium: a structurally based framework for material characterization. Philosophical Trans Royal Soci A: Math, Phys Eng Sci.

[CR30] Humphries D, Grogan J, Gaffney E (2017). Mechanical cell-cell communication in fibrous networks: the importance of network geometry. Bull Math Biol.

[CR31] Iwanaga T, Usher W, Herman J (2022) Toward SALib 2 0: Advancing the accessibility and interpretability of global sensitivity analyses. Socio-Environ Syst Modell 10.18174/sesmo.18155

[CR32] Jæger KH, Hustad KG, Cai X, Tveito A (2021). Efficient numerical solution of the emi model representing the extracellular space (e), cell membrane (m) and intracellular space (i) of a collection of cardiac cells. Front Phys.

[CR33] Jones JS, Small DM, Nishimura N (2018). In vivo calcium imaging of cardiomyocytes in the beating mouse heart with multiphoton microscopy. Front Physiol.

[CR34] Kakaletsis S, Lejeune E, Rausch M (2020) LV mechanical data. Texas Data Repository. Retrieved from https://dataverse.tdl.org/dataverse/RVMechanics10.18738/T8/KN3K9S

[CR35] Kakaletsis S, Meador WD, Mathur M, Sugerman GP, Jazwiec T, Lejeune E, Timek TA, Rausch  MK  (2021). Right ventricular myocardial mechanics: multi-modal deformation, microstructure, modeling, and comparison to the left ventricle. Acta Biomater.

[CR36] Karabelas E, Gsell MA, Haase G, Plank G, Augustin CM (2022) An accurate, robust, and efficient finite element framework with applications to anisotropic, nearly and fully incompressible elasticity. Computer Methods in Applied Mechanics and Engineering p. 394114887. Elsevier, Publisher 10.1016/j.cma.2022.114887 PMC7612621 35432634

[CR37] Kuchta M, Mardal K-A, Mortensen M (2016). On the singular Neumann problem in linear elasticity. Num Linear Algebra with Appl.

[CR38] Land S, Park-Holohan S-J, Smith NP, Dos Remedios CG, Kentish JC, Niederer SA (2017). A model of cardiac contraction based on novel measurements of tension development in human cardiomyocytes. J Mol Cell Cardiol.

[CR39] Lenarda P, Gizzi A, Paggi M (2018). A modeling framework for electro-mechanical interaction between excitable deformable cells. Eur J Mech- A/Solids.

[CR40] Li XS, Demmel JW (2003). SuperLU\_DIST: A scalable distributed-memory sparse direct solver for unsymmetric linear systems. ACM Trans Math Softw doi.

[CR41] Liang L, Jones C, Chen S, Sun B, Jiao Y (2016) Heterogeneous force network in 3D cellularized collagen networks. Phys, Biol, p 136066001 10.1088/1478-3975/13/6/066001 27779119

[CR42] Mann A, Sopher RS, Goren S, Shelah O, Tchaicheeyan O, Lesman A (2019). Force chains in cell-cell mechanical communication. J Royal Soc Interface.

[CR43] Maulik SK, Mishra S (2015) Hypertrophy to failure: What goes wrong with the fibers of the heart? Indian Heart J p. 67166 10.1016/j.ihj.2015.02.012 PMC4382541 25820056

[CR44] Nolan D, McGarry J (2016) On the compressibility of arterial tissue. Ann Biomed Eng pp. 444993-1007. Springer, Publisher 10.1007/s10439-015-1417-1 26297340

[CR45] Okada J-i, Sugiura S, Nishimura S, Hisada T (2005). Three-dimensional simulation of calcium waves and contraction in cardiomyocytes using the finite element method. Am J Physiol Cell Phys.

[CR46] Oliveira B, Sundnes J (2016) Comparison of tetrahedral and hexahedral meshes for finite element simulation of cardiac electro-mechanics. (p. 164-177). 10.7712/100016.1801.9193

[CR47] Olivetti G, Melissari M, Capasso J, Anversa P (1991). Cardiomyopathy of the aging human heart myocyte loss and reactive cellular hypertrophy. Circ Res.

[CR48] Pinali C, Kitmitto A (2014). Serial block face scanning electron microscopy for the study of cardiac muscle ultrastructure at nanoscale resolutions. J Mol Cell Cardiol.

[CR49] Qin L, Huang J, Xiong C, Zhang Y, Fang J (2007). Dynamical stress characterization and energy evaluation of single cardiac myocyte actuating on flexible substrate. Biochem Biophys Res Commun.

[CR50] Reichardt M, Neuhaus C, Nicolas J-D, Bernhardt M, Toischer K, Salditt T (2020). X-ray structural analysis of single adult cardiomyocytes: tomographic imaging and microdiffraction. Biophys J.

[CR51] Rice JJ, Wang F, Bers DM, de Tombe PP (2008). Approximate model of cooperative activation and crossbridge cycling in cardiac muscle using ordinary differential equations. Biophys J.

[CR52] Rog-Zielinska EA, Norris RA, Kohl P, Markwald R (2016) The living scar – cardiac fibroblasts and the injured heart. Trends in Molecular Medicine pp. 22299–114 10.1016/j.molmed.2015.12.006 PMC4737999 26776094

[CR53] Rossi S, Ruiz-Baier R, Pavarino LF, Quarteroni A (2012). Orthotropic active strain models for the numerical simulation of cardiac biomechanics. Int J Num Methods in Biomed Eng.

[CR54] Ruiz-Baier R, Gizzi A, Rossi S, Cherubini C, Laadhari A, Filippi S, Quarteroni A (2014). Mathematical modelling of active contraction in isolated cardiomyocytes. Math Med Biol.

[CR55] Sack K, Davies N, Guccione J, Franz T (2016). Personalised computational cardiology: patient-specific modelling in cardiac mechanics and biomaterial injection therapies for myocardial infarction. Heart Failure Rev.

[CR56] Sharafi B, Blemker SS (2011) A mathematical model of force transmission from intrafascicularly terminating muscle fibers. J Biomech pp. 44112031–2039 10.1016/j.jbiomech.2011.04.038 PMC3134549 21676398

[CR57] Sopher RS, Tokash H, Natan S, Sharabi M, Shelah O, Tchaicheeyan O, Lesman A (2018) Nonlinear elasticity of the ecm fibers facilitates efficient intercellular communication. Biophys J pp. 11571357–1370. 10.1016/j.bpj.2018.07.036 10.1016/j.bpj.2018.07.036 PMC6170818 30217380

[CR58] Stein AM, Vader DA, Weitz DA, Sander LM (2011). The micromechanics of three-dimensional collagen-i gels. Complexity.

[CR59] Stinstra J, MacLeod R, Henriquez C (2010). Incorporating histology into a 3d microscopic computer model of myocardium to study propagation at a cellular level. Ann Biomed Eng.

[CR60] Telle Å (2022). Software for the paper A cell-based framework for modeling cardiac mechanics. Zenodo.

[CR61] Telle Å, Wall ST, Sundnes J (2021) Modeling cardiac mechanics on a sub-cellular scale. A. Tveito, K.-A. Mardal, & M.E. Rognes (Eds.), Modeling excitable tissue (pp. 28–43). Cham:Springer International Publishing. 10.1007/978-3-030-61157-6_3

[CR62] Ten Eyck A, Celiker F, Lew A (2008). Adaptive stabilization of discontinuous galerkin methods for nonlinear elasticity: motivation, formulation, and numerical examples. Comput Methods Appl Mech Eng.

[CR63] Tracqui P, Ohayon J (2009). An integrated formulation of anisotropic force-calcium relations driving spatio-temporal contractions of cardiac myocytes. Philosophical Trans Royal Soc A: Math, Phys Eng Sci.

[CR64] Tracqui P, Ohayon J, Boudou T (2008). Theoretical analysis of the adaptive contractile behaviour of a single cardiomyocyte cultured on elastic substrates with varying stiffness. J Theor Biol.

[CR65] Tveito A, Jæger KH, Kuchta M, Mardal K-A, Rognes ME (2017). A cell-based framework for numerical modeling of electrical conduction in cardiac tissue. Front Phys.

[CR66] Whiteley J (2017). A preconditioner for the finite element computation of incompressible, nonlinear elastic deformations. Comput Mech.

[CR67] Xi J, Lamata P, Niederer S, Land S, Shi W, Zhuang X, Smith NP (2012). The estimation of patient-specific cardiac diastolic functions from clinical measurements. Med Image Anal.

[CR68] Yin F, Chan C, Judd RM (1996) Compressibility of perfused passive myocardium. American Journal of Physiology-Heart and Circulatory Physiology pp. 2715H1864–H1870. (Publisher: American Physiological Society Bethesda, MD) 10.1152/ajpheart.1996.271.5.H1864 8945902

[CR69] Zhang C, Gao Y (2012). Finite element analysis of mechanics of lateral transmission of force in single muscle fiber. J Biomech.

[CR70] Zhang YS, Aleman J, Arneri A, Bersini S, Piraino F, Shin SR, $$\ldots$$ Khademhosseini A (2015) From cardiac tissue engineering to heart-on-a-chip: beating challenges. Biomed Mater p. 103034006 10.1088/1748-6041/10/3/034006 PMC4489846 26065674

